# Identification and characterization of five anti-mitotic sesquiterpene lactones from *Arnica cordifolia*

**DOI:** 10.1080/13880209.2025.2610026

**Published:** 2026-01-03

**Authors:** Tanner C. Lockwood, David E. Williams, Layla Molina, Raymond J. Andersen, Roy M. Golsteyn

**Affiliations:** ^a^ Natural Product Laboratory, University of Lethbridge, Lethbridge, Canada; ^b^ Department of Earth, Ocean, Atmospheric Sciences, University of British Columbia, Vancouver, Canada

**Keywords:** Anti-mitotic, *Arnica cordifolia*, Asteraceae, Mitotic spindle, Sesquiterpene lactones

## Abstract

**Context:**

Unique ecozones, such as those in Canada, play an important role in the production of distinct natural product chemicals that help plants survive highly variable abiotic conditions and herbivory. Extracts prepared from *Arnica cordifolia* Hook. (heartleaf arnica), a North American species related to the European medicinal plant *Arnica montana* L., induce mitotic arrest in human cancer cell lines with a mitotic spindle morphology distinct from other mitotic inhibitors isolated from prairie plant species.

**Objective:**

This study aimed to identify the anti-mitotic compound(s) of *A. cordifolia*.

**Materials and Methods:**

The cytotoxic and anti-mitotic activities of *A. cordifolia* extracts and their active compounds on human cancer cells were characterized by MTT assays, light microscopy, flow cytometry, and immunofluorescence microscopy. The active compounds were isolated by bioassay-guided HPLC fractionation and identified by NMR.

**Results:**

Five anti-mitotic sesquiterpene lactones were isolated from *A. cordifolia*: three previously unidentified structures, and the known compounds aromaticin and pulchellin-2α-*O*-isovalerate. These compounds induced rounded cells positive for the mitotic marker phospho-histone H3 at concentrations of 5 µM, yet had distinct effects on mitotic spindle morphology. Furthermore, aromaticin treatment induced ubiquitin foci in cells, indicating that it may target the ubiquitin-proteasome pathway.

**Discussion and Conclusion:**

This is the first report of mitotic inhibitors from *Arnica cordifolia*. Of these five compounds, three have previously undescribed chemical structures, whereas new anti-mitotic activities have been identified for aromaticin and pulchellin-2α-*O*-isovalerate. Differences in their biological activities suggest that they possess distinct cellular targets. These findings support continued exploration of Canadian botanical species as sources of structurally diverse bioactive compounds.

## Introduction

Natural products are a major source of lead compounds in drug discovery. Although species richness is greatest in tropical and equatorial regions, the rates of speciation and diversification are highest in temperate zones such as Canada (Tietje et al. 2022; Dimitrov et al. 2023). This phylogenetic diversity correlates with increased phytochemical diversity (Defossez et al. 2021), positioning Canadian plants as a promising source of bioactive natural products.

The natural product paclitaxel (Taxol^®^), a taxane, has been isolated from the North American species *Taxus brevifolia* Nutt. (Pacific yew) (Cragg 1998). Paclitaxel arrests cells in mitosis by binding to tubulin, making it both a research tool and an important chemotherapeutic agent. Previous research from our laboratory has identified other anti-mitotic natural products from Canadian plants that span multiple chemical classes (Bosco et al. 2021; Healy Knibb et al. 2024), reinforcing their value in natural product discovery. However, Canadian botanical species remain largely underexplored by natural product science (Thornburg et al. 2018).

*Arnica cordifolia* Hook. (heartleaf arnica) is a member of the Asteraceae family native to the montane cordillera ecozone of western North America (Brouillet et al. 2010). It is closely related to the European medicinal species *Arnica montana* L., which produces anti-inflammatory sesquiterpene lactones (Lyss et al. 1998). Extracts of *A. cordifolia* display cytotoxic activity, and prior studies reported several sesquiterpene lactones from this species (Merfort and Wendisch 1993). However, little is known about the specific natural products responsible for the anti-proliferative properties of *A. cordifolia* or their cellular phenotypes.

The present work investigates the sesquiterpene lactones responsible for the anti-mitotic activity of *A. cordifolia*. Bioassay-guided isolation and structure elucidation were performed, followed by characterization of their cellular effects. These findings expand the known chemical diversity of *A. cordifolia* and add to the growing body of research exploring temperate plant species as bioactive natural products.

## Materials and methods

### Collection of plant material

Aerial parts of flowering *Arnica cordifolia* Hook. (Asteraceae) plants were collected sustainably from Porcupine Hills, Alberta, Canada at 49° 58′ 15′’ N and 114° 5′ 13′’ W, elevation approximately 1700 m in June 2022 and June 2023. Permits from the local and provincial governments were obtained prior to collection. *A. cordifolia* taxonomy was confirmed to species using botanical criteria (Moss and Packer 1983), and voucher specimens were submitted to the University of Lethbridge Herbarium as Golsteyn #420. After collection, plants were dried at 40 °C for 72 h then stored at room temperature and protected from light.

### Preparation of plant extracts

Extracts were prepared from either total or separated *A. cordifolia* aerial plant parts (leaves, flowers, stems) ground into a fine powder. For each extract, 10 g of powdered material was suspended to 10% (w/v) in either 75% (v/v) EtOH (Greenfield Global; P016EAAN; purity > 99%) in water or 100% dichloromethane (Fisher Chemical; D37-4; purity > 99.5%). Suspensions were placed on a shaker overnight at room temperature and protected from light. Suspensions were then filtered by Grade 1 Whatman paper and the solvent was evaporated at room temperature. Extracts were dissolved in MeOH (Fisher Chemical; A452–4; purity > 99.9% by GC) to 50 mg/mL and stored at −20 °C for use in subsequent experiments.

### UV-visible spectroscopy

Extracts were diluted to 1 mg/mL in MeOH, then 75 µL of each extract was loaded into separate wells of a 96-well plate. A control well was loaded with 75 µL of MeOH. The absorbance of each sample was measured from 300 to 700 nm using a Biotek Epoch microplate spectrophotometer using Gen5 software (Agilent Technologies), with a step of 2 nm between reads. Absorbance data were blanked to the MeOH control.

### Cell culture

The human cell lines HT-29 (ATCC HTB-38) and U2OS (ATCC HTB-96) were obtained from the American Type Culture Collection (ATCC) and cultivated as described previously (Kubara et al. 2012). HT-29 cells were plated at a density of 3.0 x 10^5^ cells/25 cm^2^ flask and cultured for 48 h prior to treatment. U2OS cells were plated at a density of 3.0 x 10^5^ cells/25 cm^2^ flask and cultured for 24 h prior to treatment. HT-29 cells were selected for further study due to their capacity to sustain a prolonged mitotic arrest (Gascoigne and Taylor 2008).

The compounds nocodazole (Sigma-Aldrich; M1404; purity > 99% by TLC), paclitaxel (Sigma-Aldrich; T7402; purity > 95% by HPLC), pulchelloid A (previously isolated (Bosco et al. 2021)), and MG132 (Sigma-Aldrich; M7449-1ML; purity > 90% by HPLC) were dissolved in DMSO (Sigma-Aldrich; D2438; purity > 99.9% by GC), stored at −20 °C, and used at concentrations of 500 nM, 100 nM, 5 µM, and 300 nM respectively. In not-treated cells, 0.4% (v/v) MeOH or DMSO was added as a solvent vehicle control.

### Light microscopy

Images were captured with an Infinity 1 camera with Infinity Capture imaging software (Lumenera Corporation) on an Olympus CKX41 inverted microscope. Images were processed using Adobe Photoshop (CC 25.6.0). Cells were manually scored for rounded or flat morphology and counted with ImageJ software (IJ 1.54i). At least 200 cells were counted for each treatment.

### Cell viability assay

The cytotoxicity of plant extracts was measured by the 3-(4,5-dimethylthiazol-2-yl)-2,5-diphenyltetrazolium bromide (MTT) assay (Sigma-Aldrich; M2128-1G; purity > 98%). HT-29 cells were plated at a density of 5000 cells/well in a 96-well culture plate and cultured at 37 °C for 48 h prior to treatment. After 72 h of treatment, 20 µL of MTT solution (5 mg/mL MTT in PBS (137 mM NaCl, 3 mM KCl, 100 mM Na_2_HPO_4_, 18 mM KH_2_PO_4_)) was added to each well and incubated at 37 °C for 3.5 h. The media were then removed and 150 µL of MTT solvent (4 mM HCl, 0.1% (v/v) octylphenoxypolyethoxyethanol, in isopropanol) was added to each well. Plates were placed on a shaker in the dark until all formazan crystals had dissolved, then absorbance was measured at 590 nm using a Cytation^™^ 5 Cell Imaging Multi-Mode Reader with Gen5 software (Agilent Technologies) and blanked against a control well not seeded with cells.

The concentrations of each plant extract or compound were plotted against normalized percent absorbance using GraphPad Prism software. The normalized percent absorbance was calculated as:

Normalized percent absorbance =(absorbance/DMSO absorbance) x 100

where DMSO absorbance refers to the absorbance of 0.1% (v/v) DMSO-treated cells from a control well. IC_50_ values were determined through non-linear regression (inhibitor concentration versus normalized response) using GraphPad Prism software. All treatments were tested in triplicate and independent experiments were conducted at least three times.

### Flow cytometry

HT-29 cells were seeded at a density of 0.3 × 10^5^ cells/25 cm^2^ flask and incubated at 37 °C for 48 prior to treatment. After 18 h treatment, cells were collected by trypsinization and centrifuged at 300 × g for five minutes. Cells were washed with warm PBS with 0.8% (v/v) FBS and 1 mM EDTA (Caledon Laboratories; 3460-1; purity > 95%), then resuspended to a concentration of 2.0 × 10^6^ cells/mL in cold PBS with 0.8% (v/v) FBS and 1 mM EDTA. From each suspension, 500 µL was added dropwise to 500 µL of cold 70% EtOH in water then stored at −20 °C overnight. Cells were then resuspended and 200 µL aliquots were transferred to 1.5 mL microfuge tubes. Each aliquot was centrifuged at 300 x g for five minutes, washed with PBS, centrifuged again, then resuspended in 200 µL of Muse^™^ Cell Cycle Staining Reagent for 30 min in the dark. Cells were then analyzed for DNA content using a Muse^™^ Cell Analyzer. Gating was set using a non-treated negative control and a nocodazole-treated positive control. Flow cytometry assays were performed three times.

### β-mercaptoethanol reduction assay

HT-29 cells were plated at a density of 2.0 × 10^4^ cells/well in 12-well culture plates and incubated at 37 °C for 48–72 h prior to treatment. Treatments were incubated at 37 °C for 2 h with or without 100 µM β-mercaptoethanol (MP BioMedical, 02194705-CF; purity > 98%) prior to addition to cells. After 18 h treatment, light microscopy images were taken as described above. Cells were manually scored for rounded or flat morphology. At least 200 cells were counted for each treatment.

### Immunofluorescence microscopy

HT-29 cells were plated at a density of 2.0 x 10^5^ cells/well in 6-well culture plates or 2.0 x 10^4^ cells/well in 12-well culture plates and incubated at 37 °C for 48–72 h prior to treatment. After 18 h treatment, the media were aspirated and cells were fixed with 3% (v/v) paraformaldehyde (Ted Pella; 18505; EM grade) in PBS for 20 min at room temperature. Fixation was then quenched with 50 mM NH_4_Cl (Sigma-Aldrich; A4514) in PBS for 10 min. Cells were permeabilized with 0.2% (v/v) Triton X-100 (Millipore; EM-9410) in PBS for 5 min, followed by blocking with 3% (w/v) BSA (MP Biomedicals; 810551) in PBS-T (0.1% (v/v) Tween-20 (VWR Life Science; EM-9480; purity > 96%) in PBS) for 30 min. Cells were then incubated overnight at 4 °C with anti-PH3 (Millipore; 06–570; 1:1000), anti-α-tubulin (Santa Cruz Biotechnology; sc-53030; 1:300), or anti-ubiquitin (Cell Signaling; 58395; 1:300) primary antibodies. The following day, cells were washed with PBS-T, then incubated at room temperature for 45 min with secondary antibodies Alexa Fluor^™^ 594 AffiniPure^™^ goat anti-rabbit IgG (Jackson ImmunoResearch; 111-585-003; 1:400) and Alexa Fluor^™^ 488 goat anti-rat IgG (Invitrogen; A11006; 1:200). DNA was stained with 300 nM DAPI (Invitrogen; D1306; purity > 95% by HPLC) in PBS-T for 15 min at room temperature. Cells were then imaged with a Cytation^™^ 5 Cell Imaging Multi-Mode Reader using Gen5 software (Agilent Technologies). The total number of cells (stained with DAPI) and the number of cells positive for PH3 were counted manually. Baseline mitotic spindle morphology was determined from DMSO-treated mitotic cells. A minimum of 200 cells were counted for each treatment, and three independent experiments were conducted.

### Bioassay-guided fractionation

Dried leaves (100 g) from *A. cordifolia* were extracted with MeOH (4 × 600 mL) overnight at room temperature. The combined MeOH extracts were dried and partitioned between H_2_O (400 mL) and EtOAc (3 × 100 mL). The active EtOAc extracts were combined and dried. Approximately half of the EtOAc soluble material was chromatographed on Sephadex LH20 using a 100 × 4 cm column with 80% methanol/CH_2_Cl_2_ as eluent. The fractions A-F were analyzed with the cell rounding assay and the active fraction C (205 mg) was fractionated by silica gel flash chromatography (step gradient: 19:1 hexanes/EtOAc to EtOAc to 1:9 MeOH/EtOAc, 10 g Sep-Pak (Waters Corporation)). The bioactive 1:9 EtOAc/hexanes fraction (55 mg) was then purified *via* C_18_ reversed-phase HPLC using an InertSustain 5 µm, 25 × 1 cm column with 11:9 MeCN/H_2_O as eluent and a flow rate of 2 mL/min to give pure samples of sesquiterpenes RA-312 (2.0 mg, Rt = 15.8 min) and RA-315 (4.3 mg, Rt = 37.5 min) along with a fraction containing a mixture of RA-313, RA-314-1 & 2 (Rt = 29–32 min). The mixture was separated using the same HPLC column but with 11:9 H_2_O/MeCN as eluent to give 1.9 mg of RA-313 (Rt = 62.2 min) and 2.3 mg of a 1:1 inseparable mixture of RA-314-1 & 2 (broad peak with Rt = 66.8 min). The structures of the sesquiterpenes were elucidated by analysis of standard 1D and 2D nuclear magnetic resonance (NMR) spectra and high-resolution electrospray ionization mass spectrometry (HRESIMS) (Figures 8 and 9; see also Supporting Information).

**RA-312**: Identified as aromaticin after comparison with literature values (Romo et al. 1964). Copies of the original spectra are obtainable from the corresponding author.

**RA-313**: Isolated as a clear glass; [a]^25^_D_ +20.5° (c 1.3, CH_2_Cl_2_); UV (77:33 (11:9 H_2_O/MeCN) λ_max_ 210 nm; ^13^C and ^1^H NMR, see Tables SI1 & SI2 (Supporting Information), respectively; positive ion HRESIMS [M + H]+ m/z 351.2161 (calculated for C_20_H_31_O_5_, 351.2166). Given the name pulchellin-2α-*O*-(2-methylbutyrate) in accordance with historical naming conventions (Bohlmann et al. 1985).

**RA-314-1** & **2**: Isolated as a clear glass; [a]^25^_D_ +14.5° (c 1.5, CH_2_Cl_2_); UV (11:9 H_2_O/MeCN) λ_max_ 212 nm; ^13^C and ^1^H NMR, see Tables SI1 & SI2 (Supporting Information), respectively; positive ion HRESIMS [M + H]^+^ m/z 351.2161 (calculated for C_20_H_31_O_5_, 351.2166). RA-314-1 was identified as pulchellin-2α-*O*-isovalerate after comparison with literature values (Bohlmann et al. 1985). RA-314-2 was given the name neopulchellin-2α-*O*-(2-methylbutyrate) in accordance with historical naming conventions (Bohlmann et al. 1985).

**RA-315**: Isolated as a clear glass; [a]^25^_D_ +29.5° (c 2.9, CH_2_Cl_2_); UV (11:9 MeCN/H_2_O) λ_max_ 205 nm; ^13^C and ^1^H NMR, see Tables SI1 & SI2 (Supporting Information), respectively; positive ion HRESIMS HRESIMS [M + H]+ m/z 349.2008 (calculated for C_20_H_29_O_5_, 349.2010). Given the name baileyin-2-­epi-*O*-(2-methylbutyrate) in accordance with historical naming conventions (Bohlmann et al. 1985).

The ^1^H and ^13^C NMR spectra were recorded on a Bruker AV-600 spectrometer with a 5 mm CPTCI cryoprobe.^1^H chemical shifts are referenced to the residual C_6_D_6_ (δ 7.15 ppm) and ^13^C chemical shifts are referenced to the C_6_D_6_ solvent peak (δ 128.0 ppm). Merck Type 5554 silica gel plates were used for analytical thin layer chromatography. Low- and high-resolution ESI-QIT-MS were recorded on a Bruker-Hewlett Packard 1100 Esquire–LC system mass spectrometer. Reversed-phase HPLC purifications were performed on a Waters 1525 Binary HPLC pump attached to a Waters 2998 Photodiode Array Detector. All solvents used for HPLC were Fisher HPLC grade.

### Statistical analysis

Data were analyzed using Microsoft Excel 365 and GraphPad Prism 10 software. Data were plotted as means of three independent experiments ± standard error of the means. One-way analysis of variance (ANOVA) with Tukey’s or Dunnett’s post hoc tests were used to determine statistical significance in bar graphs with the exception of β-mercaptoethanol reduction assays, where two-way ANOVA was used. Differences were considered statistically significant when *p* < 0.05.

## Results

**Figure 1.**
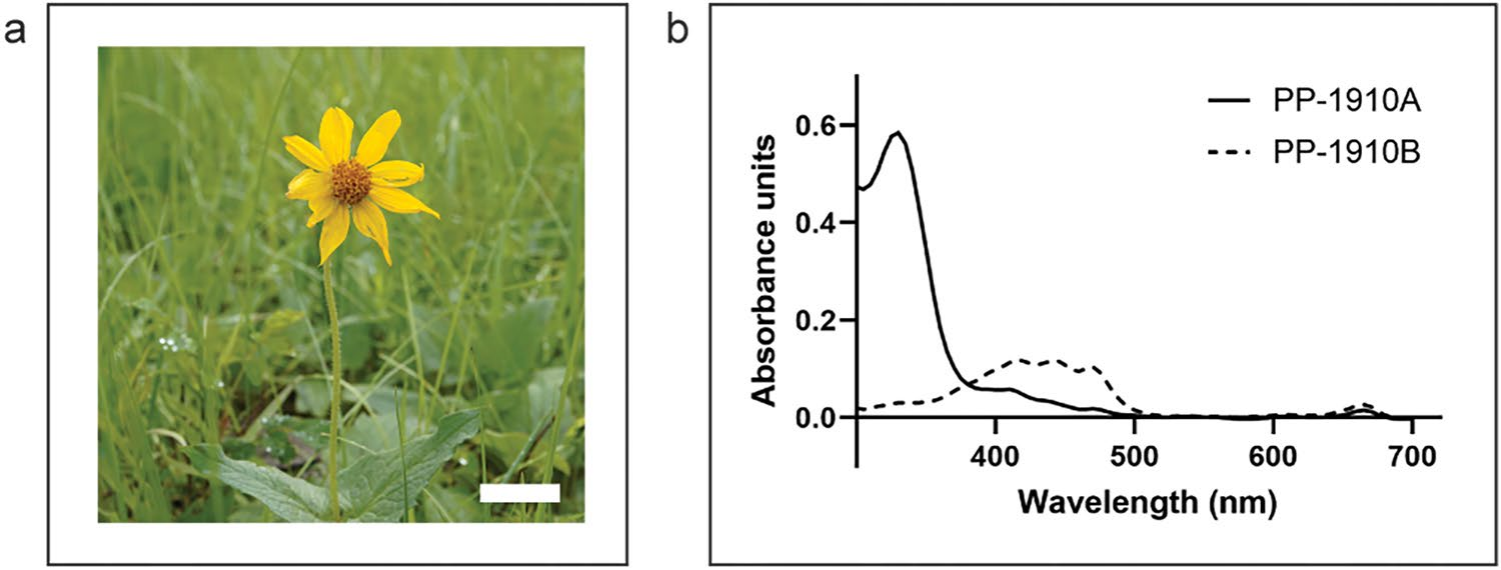
Ethanolic and dichloromethane extracts prepared from *A. cordifolia* are spectrophotometrically distinct. **a.** *A. cordifolia*. Scale bar represents 5 cm. **b.** UV/visible absorbance spectra of 75% (v/v) ethanol in water (PP-1910A) and 100% dichloromethane (PP-1910B) extracts prepared from *A. cordifolia* aerial plant material.

Aerial parts of *A. cordifolia* were collected in-bloom by sustainable methods with permission on public land in southern Alberta, Canada. The collected material was dried, then extracted with either 75% (v/v) EtOH in water (PP-1910A) or 100% dichloromethane (PP-1910B). Analysis by UV-visible spectrophotometry revealed that PP-1910A absorbed relatively strongly at 350 nm, whereas PP-1910B had relatively stronger absorbance from 380 to 480 nm, suggesting that the two extracts differed in chemical composition (Figure 1).

**Figure 2.**
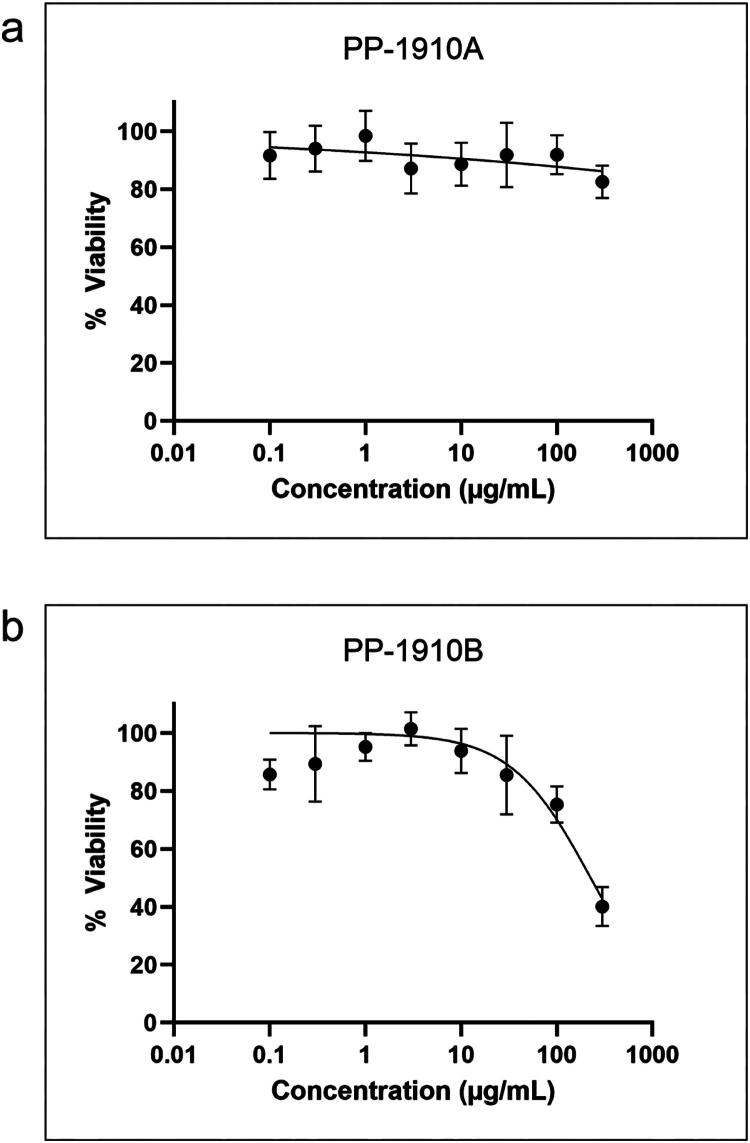
Dichloromethane extract prepared from *A. cordifolia* is cytotoxic to HT-29 cells. HT-29 cells were treated with varying concentrations of PP-1910A (**a**) or PP-1910B (**b**) for 72 h, then cell viability was determined by MTT assay. Standard errors of the means are shown.

The cytotoxicity of each extract was tested on HT-29 cells by MTT assay (Figure 2). HT-29 cells were treated with either a solvent control of DMSO (not-treated), a positive control of camptothecin (CPT), or with PP-1910A or PP-1910B at concentrations ranging from 0.1 to 300 μg/mL for 72 h, then the half-maximal inhibitory concentration (IC_50_) of each treatment was determined. CPT treatment had an IC_50_ of 36 ± 8 nM, as expected (not shown). PP-1910A treatment did not affect cell viability at any tested concentration, whereas PP-1910B treatment at 300 μg/mL, the highest concentration tested, decreased cell viability to 40%. These data revealed that extract PP-1910A was not toxic and PP-1910B was modestly toxic to HT-29 cells, and guided the selection of treatment concentrations in subsequent assays.

**Figure 3.**
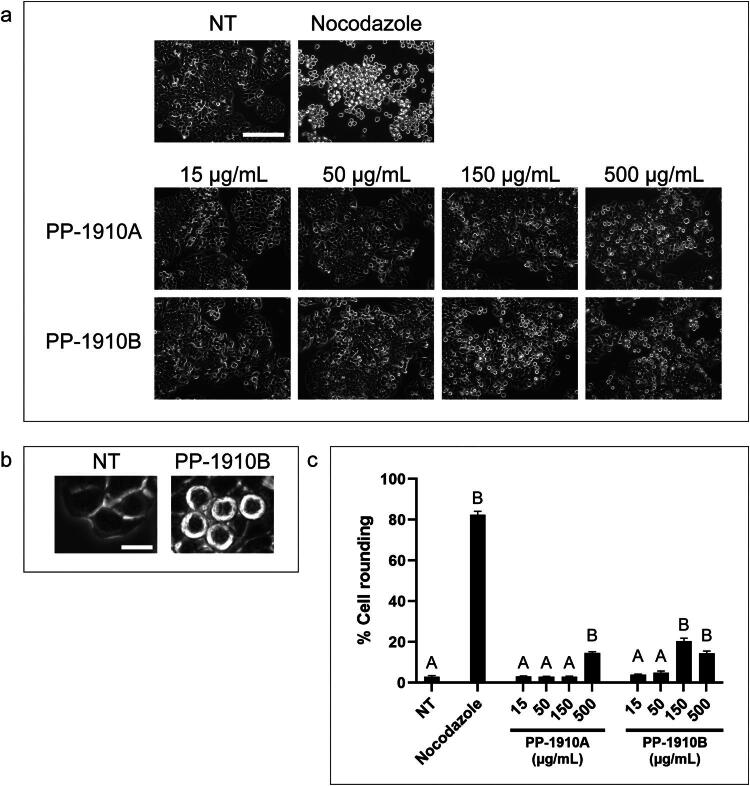
*A. cordifolia* extracts induce a rounded morphology in HT-29 cells. **a.** HT-29 cells were either not-treated (NT) or treated with nocodazole or various concentrations of PP-1910A or PP-1910B for 18 h and observed by light microscopy. Scale bar represents 50 μm. **b.** Zoomed images of not-treated cells and cells treated with 150 μg/mL PP-1910B show a rounded morphology indicative of mitosis. Scale bar represents 10 μm. **c.** The mean percentages of rounded cells after each treatment described in **a** were determined by counting cells and manually scoring them for rounded morphology. The standard errors of the means are shown and treatments that were significantly different from not-treated cells are shown by letters (*p* < 0.05).

Cells were next examined for phenotypic changes after treatment with *A. cordifolia* extracts (Figure 3). HT-29 cells were either not-treated or treated with nocodazole, which induces a mitotic arrest, or with PP-1910A or PP-1910B at a range of concentrations. After 18 h treatment, cells were observed by light microscopy and the percentage of cells with rounded morphology was determined. In not-treated HT-29 cells, 3 ± 1% exhibited a rounded morphology, whereas 82 ± 2% of nocodazole-treated cells were rounded, as expected. Treatment of HT-29 cells with PP-1910A induced 15 ± 1% cell rounding at 500 μg/mL, but did not induce cell rounding at lower concentrations. PP-1910B induced 20 ± 1% cell rounding at 150 μg/mL and 14 ± 1% cell rounding at 500 μg/mL. Additionally, a dichloromethane extract was prepared from *A. cordifolia* material collected during a different year. The percentages of rounded cells induced after treatment of HT-29 cells with the two dichloromethane extracts were not significantly different (not shown).

**Figure 4.**
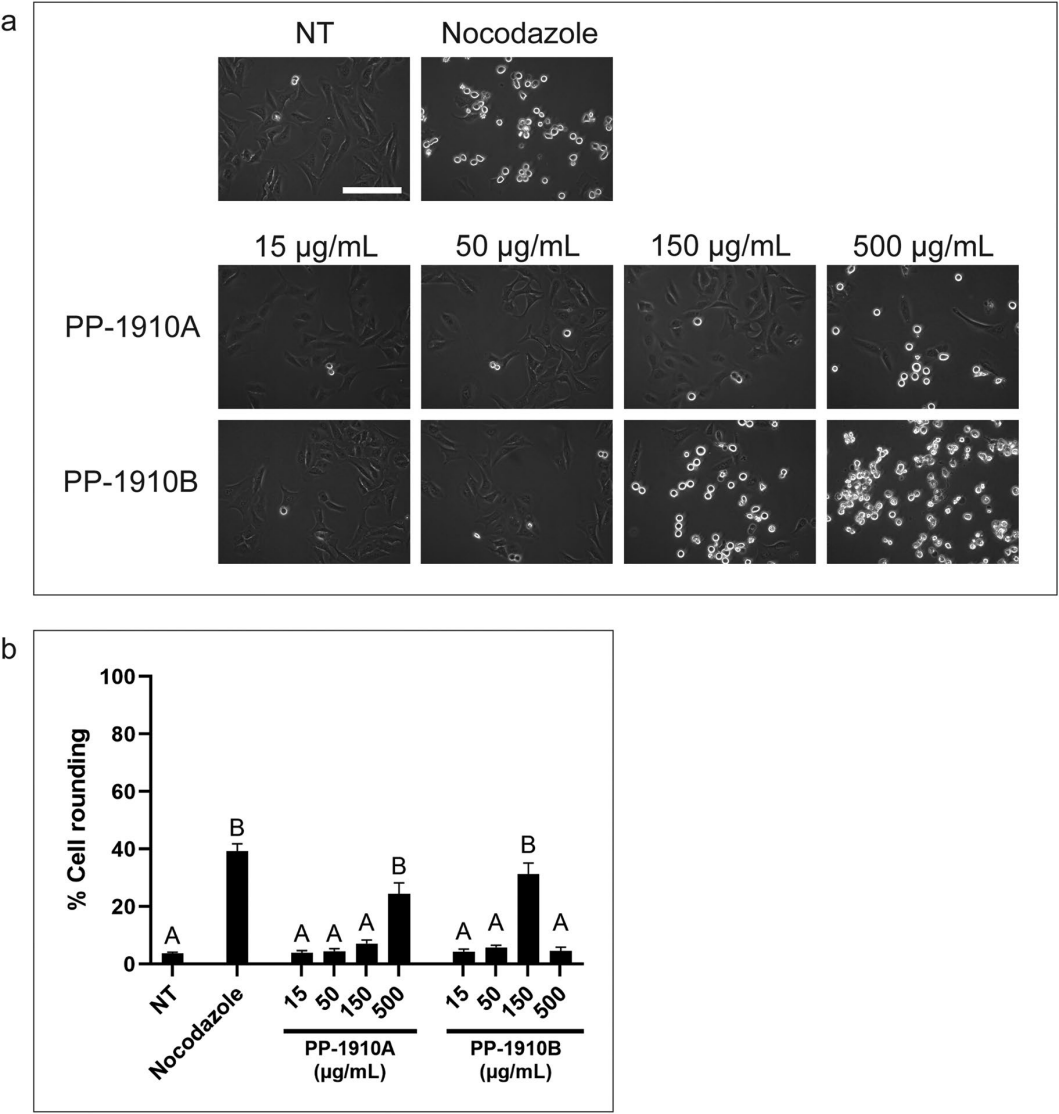
*A. cordifolia* extracts induce a rounded morphology in U2OS cells. **a.** U2OS cells were either not-treated (NT) or treated with nocodazole or various concentrations of PP-1910A or PP-1910B for 18 h and observed by light microscopy. Scale bar represents 50 μm. **b.** The mean percentages of rounded cells after each treatment described in **a** were determined. The standard errors of the means are shown and treatments that were significantly different from not-treated cells are shown by letters (*p* < 0.05).

The extracts were then applied to U2OS cells to determine whether the induction of a rounded morphology would occur in another cell line (Figure 4). Similar to HT-29 cells, not-treated U2OS cells exhibited little rounding, whereas 39 ± 3% of nocodazole-treated cells were rounded. PP-1910A induced 24 ± 4% cell rounding at 500 μg/mL in U2OS cells but did not induce cell rounding at lower concentrations. PP-1910B induced 31 ± 4% cell rounding at 150 μg/mL and was toxic at 500 μg/mL, which was consistent with the MTT assay analysis. As the dichloromethane extract PP-1910B induced cell rounding in both cell lines at lower concentrations than the ethanolic extract, it was selected for further investigation.

**Figure 5.**
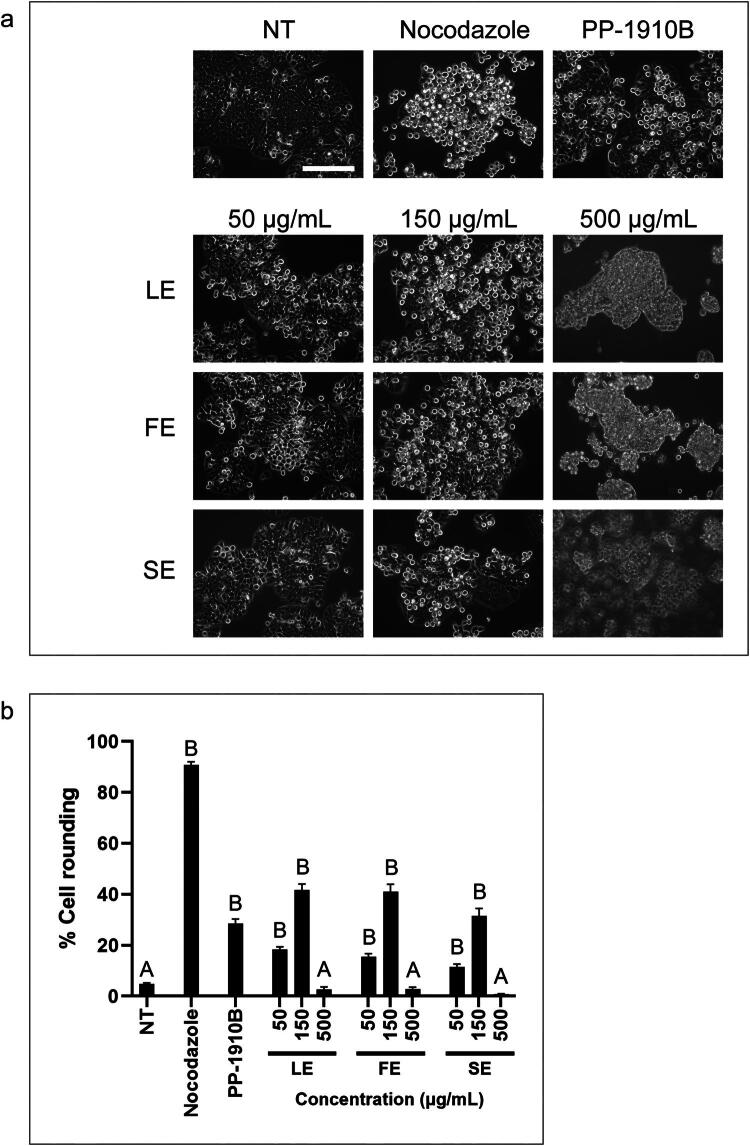
Extracts prepared from *A. cordifolia* leaves, flowers and stems induce cell rounding. **a.** HT-29 cells were either not-treated (NT) or treated with nocodazole, 150 μg/mL PP-1910B, or various concentrations of *A. cordifolia* leaf extract (LE), flower extract (FE), or stem extract (SE) for 18 h and observed by light microscopy. Scale bar represents 50 μm. **b.** The mean percentages of rounded cells from **a** were determined. The standard errors of the means are shown and treatments that were significantly different from not-treated cells are shown by letters (*p* < 0.05).

To determine whether a particular plant organ contained the active compounds that induced cell rounding, 100% dichloromethane extracts were prepared from *A. cordifolia* leaves, flowers, and stems and tested in HT-29 cells by cell rounding assay (Figure 5). Cells were either not-treated, treated with nocodazole, or treated with whole or plant organ extracts for 18 h. At 150 μg/mL, stem extract (SE) induced cell rounding similar to that of the whole extract (SE: 31 ± 3% PP-1910B: 29 ± 2%), whereas leaf extract (LE) and flower extract (FE) were more potent (LE: 42 ± 2%, FE: 41 ± 3%). These data suggest that *A. cordifolia* aerial parts, particularly leaves and flowers, contain compounds that induce cell rounding.

**Figure 6.**
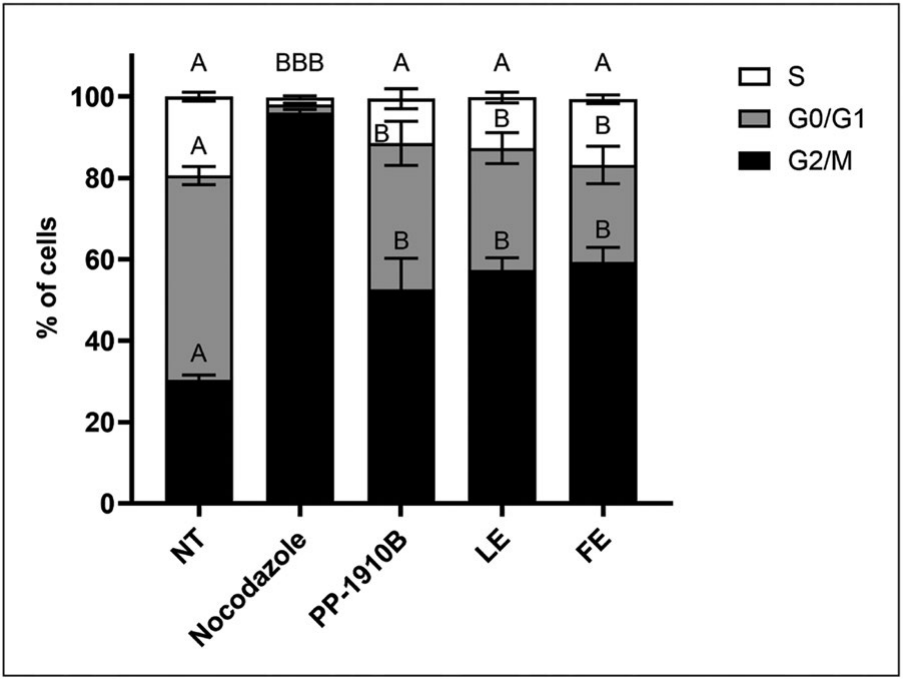
*A. cordifolia* extracts induce G2/M phase arrest. HT-29 cells were either not-treated (NT) or treated with nocodazole or 150 μg/mL PP-1910B, leaf extract (LE), or flower extract (FE) for 18 h and analyzed for DNA content by flow cytometry. G0/G1 represents the 2 N fraction and G2/M represents the 4 N fraction. The standard errors of the means are shown and fractions that were significantly different from not-treated cells are shown by letters (*p* < 0.05).

As a rounded cell morphology is a feature of cells arrested in mitosis (Lancaster et al. 2013), the rounded cells observed by light microscopy after PP-1910B treatment were examined by flow cytometry to determine if they were arrested in the cell cycle. HT-29 cells were either not-treated, treated with nocodazole, or treated with 150 μg/mL PP-1910B, LE or FE for 18 h, then fixed and examined for DNA content by flow cytometry (Figure 6). Not-treated cells had a phase distribution of 50 ± 2% in G0/G1, 19 ± 1% in S, and 30 ± 1% in G2/M phases, whereas 96 ± 1% of nocodazole-treated cells were in G2/M phase. The phase distributions of cells treated with PP-1910B (G0/G1 = 36 ± 5%, *S* = 11 ± 2%, G2/*M* = 53 ± 8%), LE (G0/G1 = 30 ± 4%, *S* = 12 ± 1%, G2/*M* = 58 ± 3%) and FE (G0/G1 = 24 ± 5%, *S* = 16 ± 1%, G2/*M* = 59 ± 4%) were significantly different from those of the not-treated or nocodazole-treated cell populations. These data indicate that cells treated with *A. cordifolia* extracts arrest in G2/M phase of the cell cycle.

**Figure 7.**
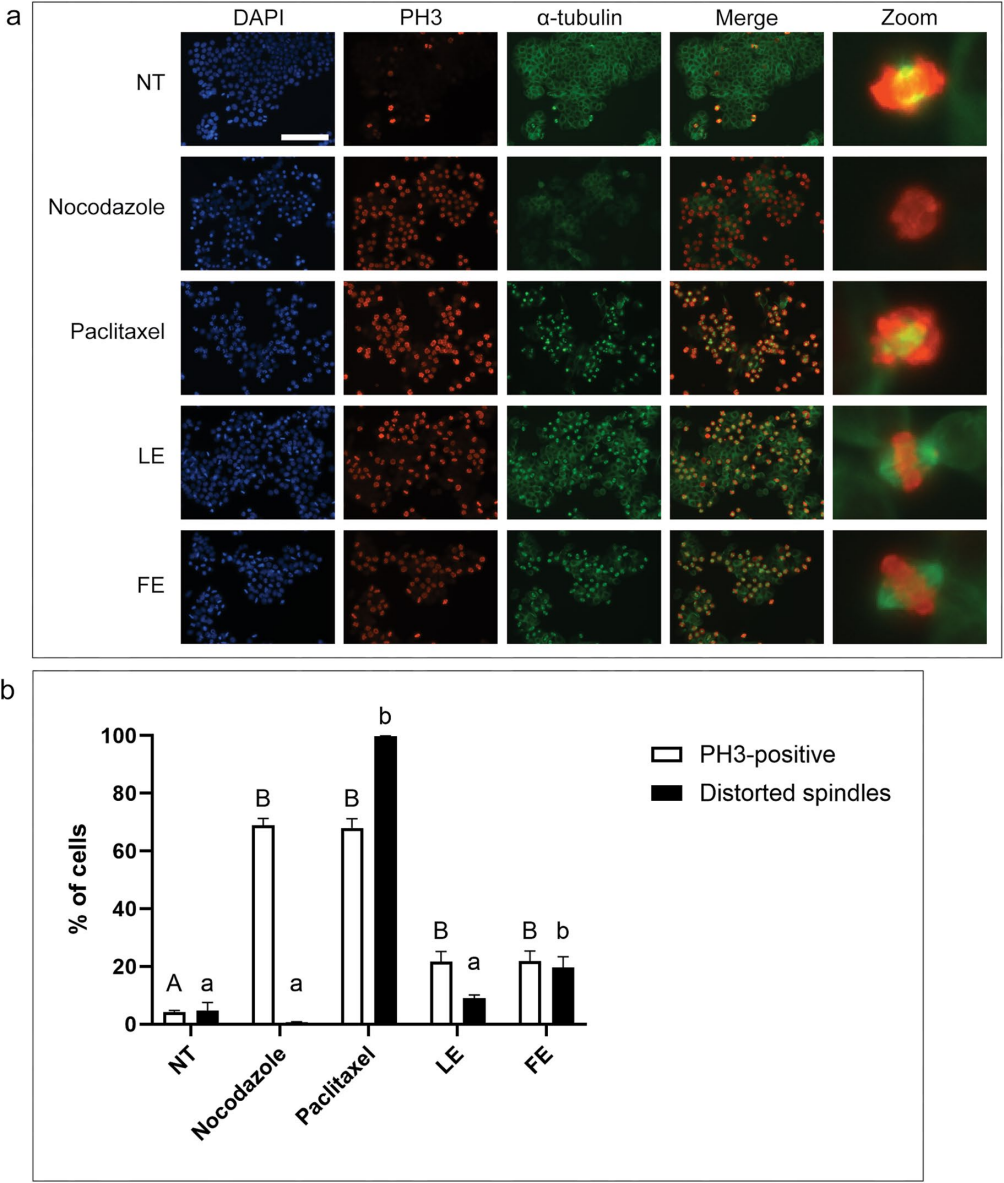
Cells treated with *A. cordifolia* extracts contain phosphorylated histone H3, but these extracts have differing effects on the mitotic spindle. **a.** HT-29 cells were either not-treated (NT) or treated with nocodazole, paclitaxel, or 150 μg/mL leaf extract (LE) or flower extract (FE) for 18 h and stained with DAPI (blue) to detect DNA, anti-phospho-histone H3 antibodies (PH3, red) and anti-α-tubulin antibodies (green). The merge column shows both PH3 and α-tubulin staining. Cells were observed by immunofluorescence microscopy. Scale bar represents 50 μm. **b.** The mean percentages of cells with PH3 staining and of mitotic spindles with distorted appearances after each treatment described in **a** were determined. Mitotic spindles of PH3-positive cells were manually scored for distorted mitotic spindle morphology in reference to NT cells. The standard errors of the means are shown and treatments that were significantly different from not-treated cells are shown by letters (*p* < 0.05).

To distinguish between a G2 or M phase arrest, immunofluorescence microscopy with phospho-Ser10 histone H3 (PH3) antibodies to identify mitotic cells and α-tubulin antibodies was used to examine mitotic spindle organization (Figure 7). Two tubulin inhibitors were used as positive controls for an M phase arrest: nocodazole, which depolymerizes microtubules, and paclitaxel, which stabilizes microtubules. Cells were either not-treated, treated with one of two tubulin inhibitors, or treated with LE or FE for 18 h. The mean percentages of PH3-positive cells were 4 ± 1% for not-treated cells, 69 ± 3% for nocodazole-treated cells and 68 ± 4% for paclitaxel-treated cells, which confirmed the accumulation of mitotic cells after nocodazole or paclitaxel treatment relative to not-treated cells. Strikingly, 22 ± 4% of cells treated with LE and 22 ± 4% of cells treated with FE were PH3-positive. These data confirmed that the cell rounding observed after *A. cordifolia* extract treatment represented cells arrested in mitosis.

Immunofluorescence microscopy also facilitated the comparison of α-tubulin organization between not-treated cells, cells treated by known tubulin toxins, and cells treated by LE or FE (Figure 7). Not-treated cells displayed a normal range of mitotic tubulin structures, including bipolar mitotic spindles. Nocodazole-treated cells lacked mitotic spindles, whereas paclitaxel-treated cells showed intensely stained and distorted spindle structures. In cells treated with FE, 20 ± 5% of mitotic cells showed distorted mitotic spindle structures that were distinguishable from those of paclitaxel-treated cells. Strikingly, only 9 ± 1% of LE-treated cells showed a distorted mitotic spindle; this was not significantly different from the percentage of distorted spindles detected in not-treated cells (5 ± 4%). The presence of mitotic cells with both undistorted spindles (LE) and distorted spindles (FE) suggested that *A. cordifolia* contained several distinct anti-mitotic compounds, which may induce arrest by distinct mechanisms. This result prompted the isolation of the anti-mitotic compounds from the leaves of *A. cordifolia.*

**Figure 8.**
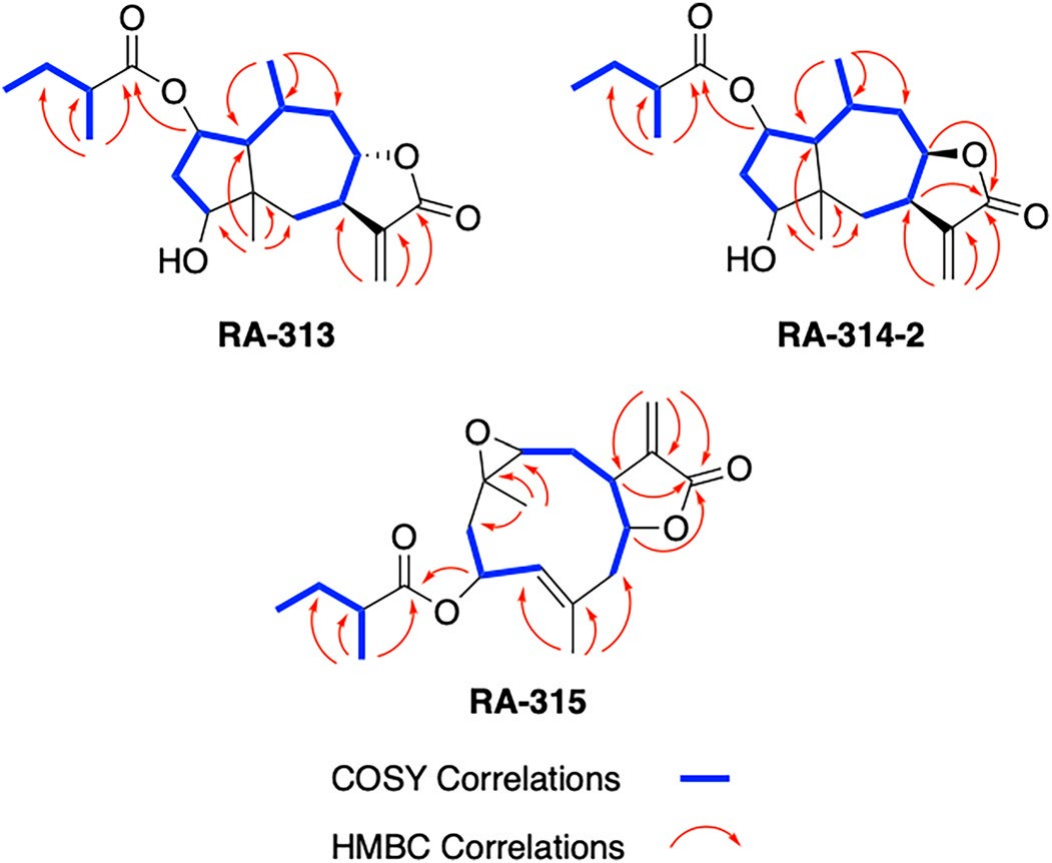
COSY and HMBC correlations used to assign the constitutions of RA-313, RA-314, and RA-315.

**Figure 9.**
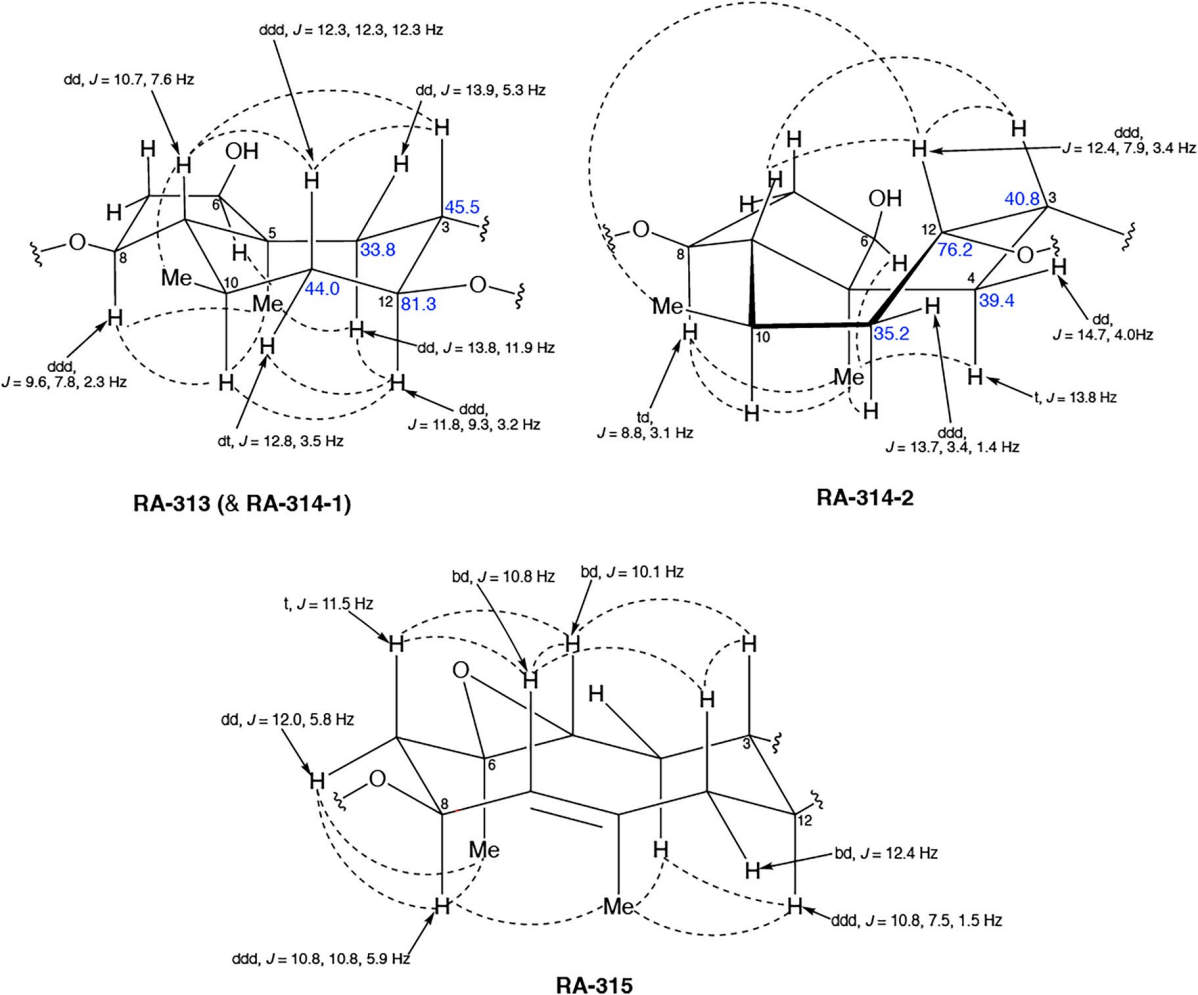
Unambiguous tROESY correlations (dashed lines) and *J* couplings used to assign the relative configurations of RA-313, RA-314-1, RA-314-2 and RA-315 along with a comparison of the ^13^C NMR assignments (ppm) (blue) about the trans/cis C-3/C-12 bridge junction in RA-313 (RA-314-1) and RA-314-2.

To accomplish this, the cell rounding assay was used to direct the bioassay-guided fractionation of *A. cordifolia* leaf material. Through successive rounds of fractionation and screening, three sesquiterpene lactones were isolated in pure form (RA-312, RA-313, RA-315), and two additional sesquiterpene lactones (RA-314-1 and RA-314-2) were co-purified as a 1:1 mixture, referred to collectively as RA-314 (Table 1). As RA-314-1 and RA-314-2 were not separated, all biological activity data reported for RA-314 reflect the effects of this unresolved mixture. Of these, three (RA-313, pulchellin-2α-*O*-(2-methylbutyrate); RA-314-2, neopulchellin-2α-*O*-(2-methylbutyrate); and RA-315, baileyin-2-epi-*O*-(2-methylbutyrate)) were previously undescribed structures, whereas RA-312 and RA-314-1 were identified as aromaticin (Romo et al. 1964) and pulchellin-2α-*O*-isovalerate (Bohlmann et al. 1985) by comparison with literature values. Structures were elucidated by detailed analysis of the 1D and 2D NMR spectra along with the high-resolution electrospray ionization mass spectra (HRESIMS) (Figures 8 and 9; see also Figure SI1, Tables SI1 and SI2, and Pages SI5-SI31, Supporting Information). Previously undescribed compounds were given names in accordance with historical naming conventions (Bohlmann et al. 1985).

**Table 1.** Comparison of the structural features and biological activities of sesquiterpene lactones isolated from *A. cordifolia.*

Feature	RA-312	RA-313	RA-314-1	RA-314-2	RA-315
Structure	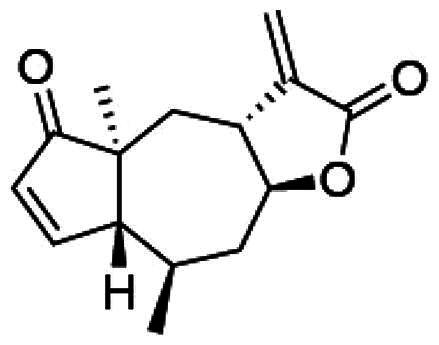	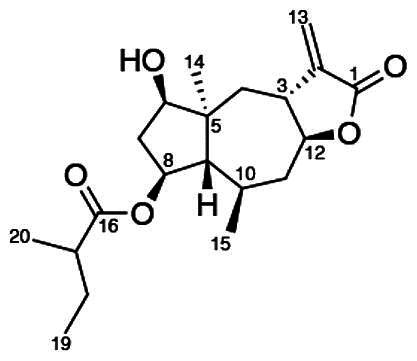	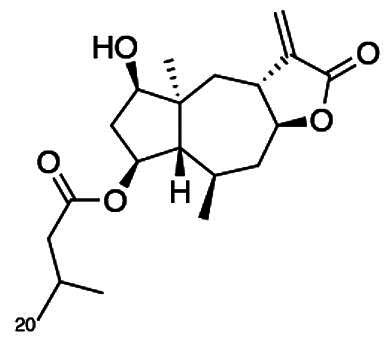	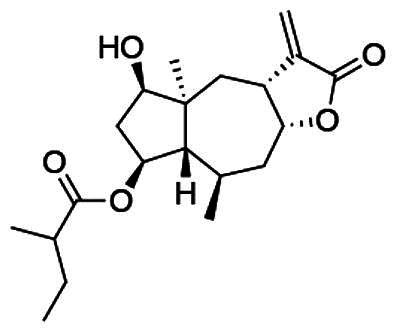	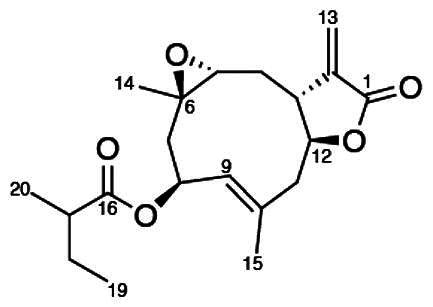
Identity	Aromaticin	Pulchellin-2α-*O*-(2-methylbutyrate)	Pulchellin-2α-*O*-isovalerate	Neopulchellin-2α-*O*-(2-methylbutyrate)	Baileyin-2-epi-*O*-(2-methylbutyrate)
Molecular mass (Da)	246.31	350.46	350.46	350.46	348.44
Class	Pseudoguaianolide	Pseudoguaianolide	Pseudoguaianolide	Pseudoguaianolide	Germacranolide
IC_50_ (μM)	2.7 ± 0.3	3.5 ± 0.3	4.2 ± 0.7		3.1 ± 0.4
Cell rounding (%)	24 ± 2%	48 ± 4%	35 ± 2%		29 ± 1%
Reduction by BME	**+**	**+**	**+**		**+**
Mitotic spindle distortion	**–**	**+**	**+**		**–**
Altered ubiquitin staining	**+**	**–**	**–**		**–**

IC_50_ values were determined by the MTT assay. All other biological activities represent a treatment concentration of 5 μM on HT-29 cells.

**Figure 10.**
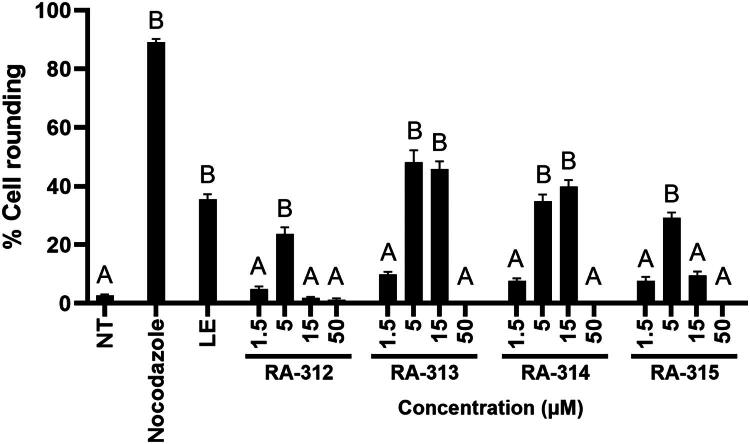
Isolated sesquiterpene lactones induce a rounded morphology in HT-29 cells. HT-29 cells were either not-treated (NT) or treated with nocodazole or various concentrations of RA-312, RA-313, RA-314, or RA-315 for 18 h and observed by light microscopy, then the mean percentages of rounded cells after each treatment were determined. The standard errors of the means are shown and treatments that were significantly different from not-treated cells are shown by letters (*p* < 0.05).

The cytotoxicity of each isolated compound was tested on HT-29 cells by MTT assay (Table 1; see also Figure SI2, Supporting Information). The IC_50_ values were within the range of 2–4 μM for all compounds. The capacity of each compound to induce cell rounding was determined by phenotypic assay. HT-29 cells were either not-treated, treated with nocodazole, or treated with increasing concentrations of each isolated compound for 18 h, then examined by light microscopy (Figure 10). Not-treated cells exhibited little rounding, whereas nearly all nocodazole-treated cells were rounded. RA-312 induced 24 ± 2% cell rounding at 5 μM and was toxic at 15 μM. RA-313 induced 48 ± 4% cell rounding at 5 μM and 46 ± 2% cell rounding at 15 μM. RA-314 induced 35 ± 2% cell rounding at 5 μM and 40 ± 1% cell rounding at 15 μM. RA-315 induced 29 ± 1% cell rounding at 5 μM and was toxic at 15 μM. None of the compounds induced cell rounding at 1.5 μM and all were toxic at 50 μM.

**Figure 11.**
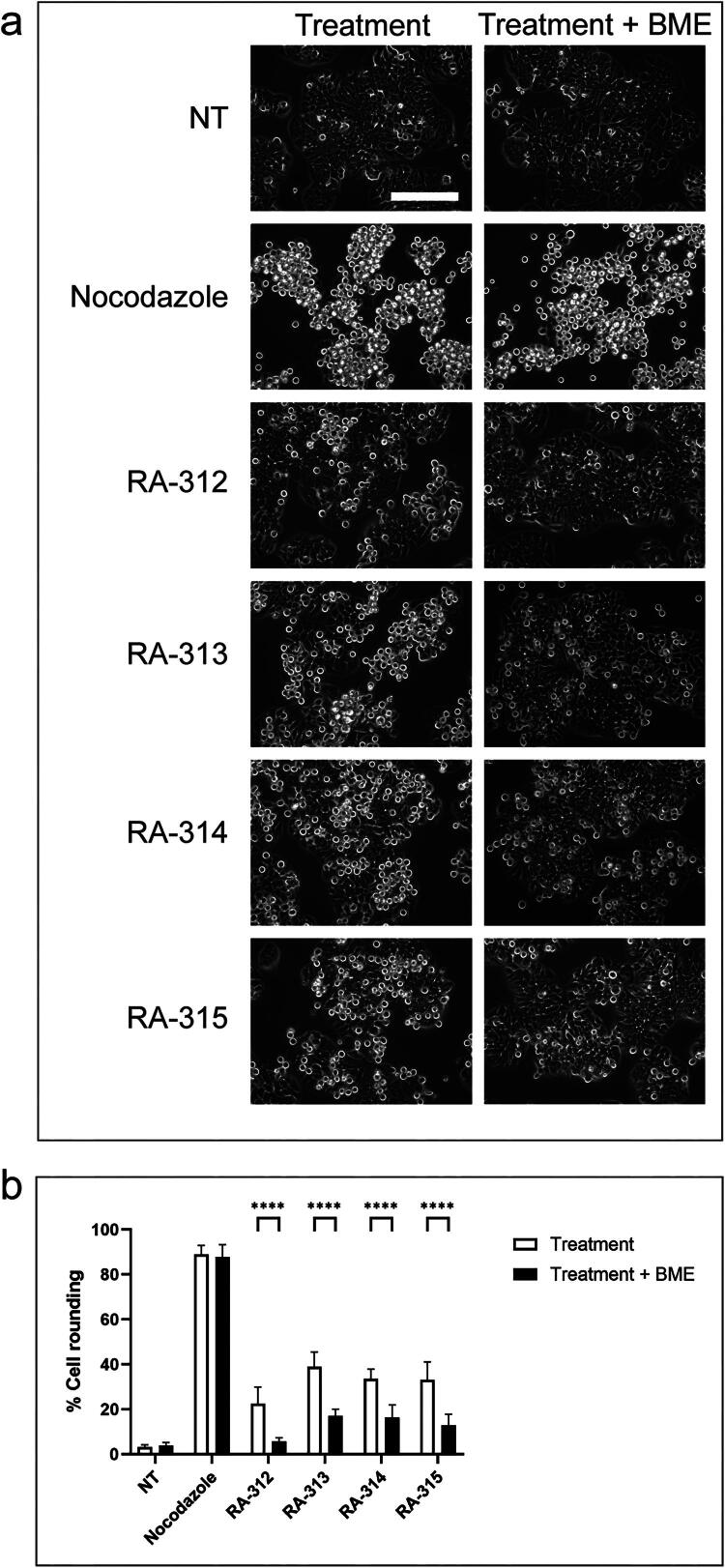
β-mercaptoethanol inhibits the anti-mitotic activity of isolated sesquiterpene lactones. **a.** Treatments of vehicle control (not-treated, NT), nocodazole, and 5 μM RA-312, RA-313, RA-314, and RA-315 were incubated at 37 °C for 2 h with or without 100 µM β-mercaptoethanol prior to addition to HT-29 cells. Cells were observed by light microscopy. Scale bar represents 50 μm. **b.** The mean percentages of rounded cells after each treatment described in **a** were determined. The standard errors of the means are shown and treatments that were significantly different from not-treated cells are shown by asterisks (*p* < 0.05).

Although the isolated compounds were structurally distinct, it was noted that each contained an α-methylene-γ-lactone moiety. To determine if the methylene function of this moiety was essential to the mitotic activity of each compound, each was incubated with β-mercaptoethanol (BME) (Figure 11). Media containing either vehicle control, nocodazole or each isolated compound were incubated for 2 h with or without 100 μM BME before their addition to HT-29 cells. Cells were examined for rounded morphology after 18 h of treatment. Both not-treated cells and nocodazole-treated cells showed no significant difference in cell rounding in the presence or absence of BME, as expected. All isolated compounds induced significantly less cell rounding after BME incubation, indicating that the α-methylene-γ-lactone moiety was essential for the anti-mitotic activities of these compounds.

**Figure 12.**
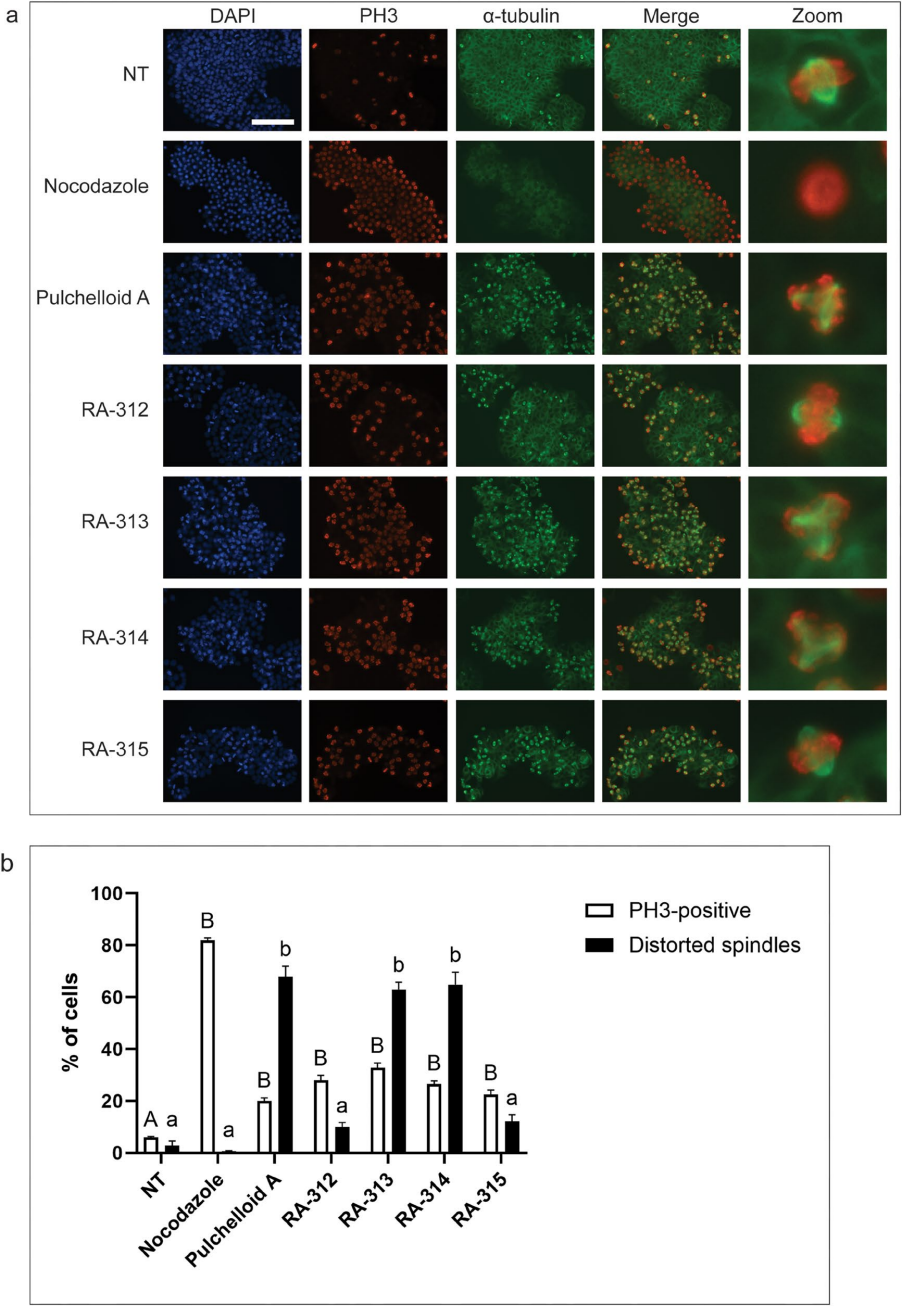
Cells treated with isolated sesquiterpene lactones contain phosphorylated histone H3, but these compounds have differing effects on the mitotic spindle. **a.** HT-29 cells were either not-treated (NT) or treated with nocodazole, pulchelloid A, or 5 μM RA-312, RA-313, RA-314, or RA-315 for 18 h and stained with DAPI (blue) to detect DNA, anti-phospho-histone H3 antibodies (PH3, red) and anti-α-tubulin antibodies (green). The merge column shows both PH3 and α-tubulin staining. Cells were observed by immunofluorescence microscopy. Scale bar represents 50 μm. **b.** The mean percentages of cells with PH3 staining and of mitotic spindles with distorted appearances after each treatment described in **a** were determined. Mitotic spindles of PH3-positive cells were manually scored for distorted mitotic spindle morphology in reference to NT cells. The standard errors of the means are shown and treatments that were significantly different from not-treated cells are shown by letters (*p* < 0.05).

To determine whether the isolated compounds would affect mitotic spindle organization in a similar manner as we had observed by the LE and FE treatments, cells were either not-treated, treated with nocodazole, treated with the anti-mitotic sesquiterpene lactone pulchelloid A (Bosco et al. 2021), or treated with the isolated compounds for 18 h and observed by immunofluorescence microscopy. Not-treated cells displayed few PH3-positive cells with the normal range of mitotic tubulin structures, including bipolar mitotic spindles (Figure 12). Nocodazole-treated cells were largely PH3-positive and lacked mitotic spindles, whereas 68 ± 4% of pulchelloid A-treated cells that were PH3-positive contained a distorted mitotic spindle, as expected. Mitotic spindle distortion was observed in PH3-positive cells after treatment with RA-313 (63 ± 3%) and RA-314 (65 ± 5%). In contrast, RA-312 induced only 10 ± 2% distorted spindles, and RA-315 induced 12 ± 3% distorted spindles. These data demonstrate that *A. cordifolia* contains multiple anti-mitotic sesquiterpene lactones that induce mitotic arrest by at least two distinct mechanisms.

**Figure 13.**
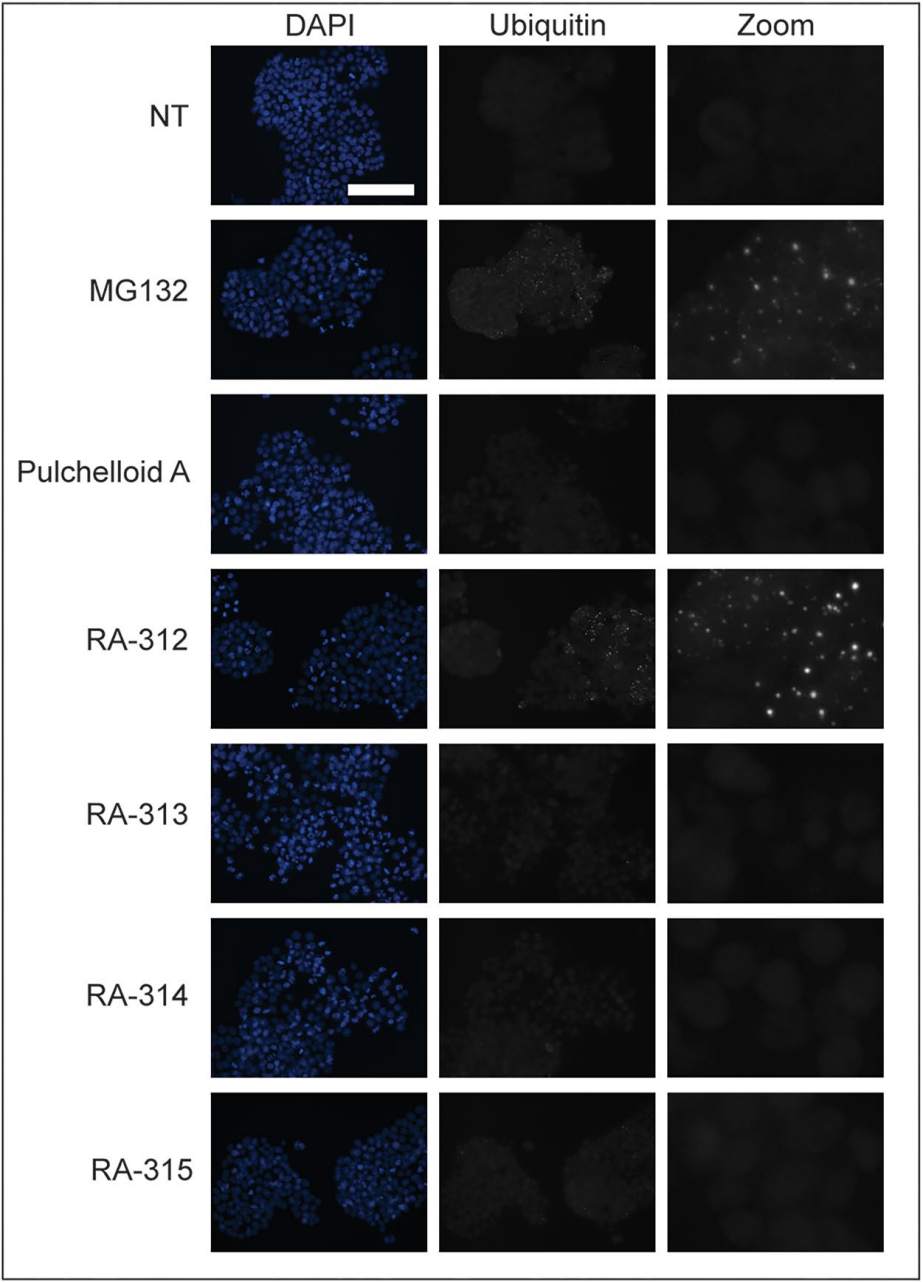
Cells treated with RA-312 show punctate ubiquitin staining. HT-29 cells were either not-treated (NT) or treated with MG132, pulchelloid A, or 5 μM RA-312, RA-313, RA-314, or RA-315 for 18 h and stained with DAPI (blue) to detect DNA and anti-ubiquitin antibodies (gray). The merge column shows both stains. Cells were observed by immunofluorescence microscopy. Scale bar represents 50 μm.

As inhibition of protein degradation can arrest mitotic cells at metaphase (Wójcik et al. 1996), it was possible that the isolated compounds targeted the ubiquitin-proteasome system. To investigate this, immunofluorescence microscopy was used to observe ubiquitin, a key regulatory protein for protein degradation, within treated cells (Figure 13). HT-29 cells were either not-treated or treated with the proteasome inhibitor MG132, pulchelloid A, or the isolated compounds for 18 h, then fixed and stained with anti-ubiquitin antibodies. Not-treated cells displayed faint and diffuse ubiquitin staining, whereas MG132-treated cells contained many conspicuous ubiquitin foci. Cells treated with pulchelloid A, RA-313, RA-314, or RA-315 showed diffuse staining resembling that of not-treated cells. Strikingly, ubiquitin foci were present in cells treated with RA-312. This phenotype suggests that RA-312 may interfere with the ubiquitin-proteasome system, Additional biochemical assays will be required to determine whether RA-312 directly impacts protein degradation pathways. This activity distinguished it from RA-315, which was otherwise biologically similar by inducing mitotic arrest without mitotic spindle distortion.

## Discussion

In this study, five anti-mitotic sesquiterpene lactones were isolated from the North American botanical species *Arnica cordifolia*: three previously undescribed structures, and the known compounds aromaticin and pulchellin-2α-*O*-isovalerate. *A. cordifolia* is an herbaceous perennial of the Asteraceae family and its native range includes the montane cordillera ecozone of Alberta, Canada (Brouillet et al. 2010). It is related to the European medicinal plant *Arnica montana* L., which produces anti-inflammatory pseudoguaianolide sesquiterpene lactones including helenalin and dihydrohelenalin (Lyss et al. 1998). The Asteraceae species *Gaillardia aristata* and *Hymenoxys richardsonii* from temperate zones have been previously shown to harbor sesquiterpene lactones that arrest human cells in mitosis with distorted spindles (Bosco et al. 2021; Molina et al. 2021). During a screening program of our botanical extract library, we observed that *A. cordifolia* extracts induced a mitotic arrest without spindle distortion. Although three sesquiterpene lactones—carabrone, graveolide, and 2,3-dihydroaromaticin—have been previously isolated from *A. cordifolia* (Merfort and Wendisch 1993) and found to be cytotoxic to several cancer cell lines (Lee et al. 2002; Zheng et al. 2013), none of these compounds were reported to induce mitotic arrest. This study describes the compounds responsible for the anti-mitotic activity of *A. cordifolia* and in each case is the first to characterize the mitotic arrest phenotypes they induce.

Of the five active compounds isolated in this study, two were previously described. Aromaticin (RA-312) is a pseudoguaianolide sesquiterpene lactone reported to be cytotoxic to multiple cancer cell lines (Cheng et al. 2012; Yu et al. 2019). A previous study reported that *A. cordifolia* contains graveolide, a compound that differs from aromaticin solely by possessing a cyclopentanone ring instead of a cyclopentenone ring, and its stereoisomer 2,3-dihydroaromaticin, but did not report aromaticin as a constituent (Merfort and Wendisch 1993). As the cyclopentenone ring of aromaticin contains an α,β-unsaturated carbonyl, it may serve as an active site in addition to the α-methylene-γ-lactone (Schmidt 2006). Pulchellin-2α-*O*-isovalerate (RA-314-1) is a pseudoguaianolide sesquiterpene lactone previously isolated from *Centipeda minima* (L.) A.Braun & Asch (Wu et al. 2012), which also contains the anti-mitotic sesquiterpene lactones 6-*O*-angeloylplenolin (Liu et al. 2011) and arnicolide D (Liu et al. 2019). These two compounds have been reported to induce G2/M phase arrest in multiple cell lines at low micromolar doses, similar to that of pulchellin-2α-*O*-isovalerate in this study. However, previous research on pulchellin-2α-*O*-isovalerate has only reported anti-angiogenic activity (Huang et al. 2016).

This study determined the chemical structures and biological activities of three previously unidentified sesquiterpene lactones: pulchellin-2α-*O*-(2-methylbutyrate) (RA-313), neopulchellin-2α-*O*-(2-methylbutyrate) (RA-314-2), and baileyin-2-epi-*O*-(2-methylbutyrate) (RA-315). Pulchellin-2α-*O*-(2-methylbutyrate) and pulchellin-2α-*O*-isovalerate (RA-314-1) differed solely by the arrangement of the ester functionality. Neopulchellin-2α-*O*-(2-methylbutyrate) was a stereoisomer of pulchellin-2α-*O*-(2-methylbutyrate), differing at the α-methylene-γ-lactone. Because pulchellin-2α-*O*-isovalerate and neopulchellin-2α-*O*-(2-methylbutyrate) were not purified from each other, the biological activities observed for RA-314 likely represent a composite response. Further separation and individual testing will be necessary to determine the specific contribution of each isomer to the mitotic phenotypes observed. Further investigation of these compounds may clarify if the stereochemistry of the α-methylene-γ-lactone ring is related to their anti-mitotic activities. Baileyin-2-epi-*O*-(2-methylbutyrate) was the most structurally distinct compound, being a germacranolide sesquiterpene lactone and possessing a C4-C5 epoxide group in contrast to the four pseudoguaianolides. Epoxide groups are a feature of some biologically active sesquiterpene lactones, such as the C4-C5 epoxide in parthenolide (Kreuger et al. 2012). Like the cyclopentenone ring of aromaticin, this epoxide group may serve as a second active site for interacting with nucleophiles (Schmidt 2006).

The isolation and characterization of the active compounds of *A. cordifolia*, especially the germacranolide baileyin-2-epi-*O*-(2-methylbutyrate) (RA-315), highlights an emerging theme that the biological activities of sesquiterpene lactones are congruent. Whereas over 6000 sesquiterpene lactones are described (Adekenov 2017), they share both common and distinct chemical features. In the BME reduction assay (Figure 11), each compound had reduced cell rounding activity after BME pretreatment, indicating that the shared electrophilic lactone functionality is likely responsible for activity. This result agrees with the reported BME sensitivity of other anti-mitotic sesquiterpene lactones including pulchelloid A (Healy Knibb et al. 2024) and psilostachyin A (Sturgeon et al. 2005). By contrast, the differences in non-lactone features, such as the pseudoguaianolide and germacranolide cyclic groups, may correspond with distinct protein targets.

This study provides evidence that several of the sesquiterpene lactone structures isolated possess similar biological activities. Only a small number of sesquiterpene lactones are reported to have anti-mitotic activity (Bosco and Golsteyn 2017; Teng et al. 2025). Of these, mitotic spindle distortion is induced by pulchelloid A (Bosco et al. 2021), hymenoratin (Molina et al. 2021), 6-*O*-angeloylplenolin (Liu et al. 2011), and psilostachyins A and C (Sturgeon et al. 2005). In comparison to the compounds isolated in this study, the structures and mitotic arrest phenotypes of RA-313 and the RA-314 mixture were most similar to those of pulchelloid A and hymenoratin: each are pseudoguaianolide sesquiterpene lactones with α-methylene-γ-lactone functionalities that induce approximately 30-40% mitotic arrest with 60-80% mitotic spindle distortion. By contrast, aromaticin and RA-315 may represent a different group of anti-mitotic sesquiterpene lactones in that they induce mitotic arrest without distortion of the mitotic spindle. Additionally, these two compounds were distinguishable from one another through their differing effects on ubiquitin organization. The ubiquitin-proteasome pathway is required for the temporal regulation of mitosis, including control of cyclins, cyclin-dependent kinases, cyclin-dependent kinase inhibitors, and mitotic proteins such as securin and Aurora kinases (Zou and Lin 2021). RA-315 treatment did not affect ubiquitin staining, whereas aromaticin treatment induced punctate staining. Other sesquiterpene lactones are also reported to affect ubiquitin pathways, including parthenolide and costunolide (Li et al. 2020), arteannuin B (Chen et al. 2024), 1β-hydroxyalantolactone (Liu et al. 2014), and 2α,6α-diacetoxy-4β-hydroxy-11(13)-pseudoguaien-12,8α-olide (Qi et al. 2022).

The identification of five structurally distinct sesquiterpene lactones from *Arnica cordifolia* highlights the chemodiversity that can arise in plant species adapted to highly variable environments (Defossez et al. 2021). The montane cordillera ecozone experiences wide temperature ranges and other abiotic stresses which may contribute to the production of bioactive secondary metabolites by *A. cordifolia* and other botanical species (Ramakrishna and Ravishankar 2011). Such conditions are known to select for structurally diverse and functionally specialized natural products (Gross et al. 2024). These findings underscore the chemical potential of underexplored Canadian botanical species as sources of novel scaffolds for probing cell biology and modulating cellular pathways.

Through the pursuit of a unique mitotic arrest phenotype, our study has contributed three previously unidentified mitotic inhibitors and attributed anti-mitotic activity to two known natural products. These discoveries underscore the value in investigating botanical species that flourish in ecosystems under highly variable abiotic conditions. These compounds broaden our understanding of the structure–function relationships of anti-mitotic natural products and represent promising candidates for future mechanistic and preclinical investigations.

## Supplementary Material

Supporting_Information_Rev1_1_1.docx

## Data Availability

The data that support the findings of this study are available from the corresponding author, RMG, upon request.
