# Supplementary material for: Identification and characterization of five anti-mitotic sesquiterpene lactones from Arnica cordifolia
Source: Pharm Biol. 2026 Jan 3;64(1):185–204. doi: 10.1080/13880209.2025.2610026 (PMC12777910; doi:10.1080/13880209.2025.2610026)
Supplement: Supporting_Information_Rev1_1_1.docx [file IPHB_A_2610026_SM7120.docx]

Supporting Information

**Identification and characterization of five anti-mitotic sesquiterpene lactones from *Arnica cordifolia***

Tanner C. Lockwood^1^, David E. Williams^2^, Layla Molina^1^, Raymond J. Andersen^2^ and Roy M. Golsteyn^1*^

1. Natural Product Laboratory, University of Lethbridge, 4401 University Drive W, Lethbridge, AB, T1K 3M4, Canada
2. Departments of Chemistry and Earth, Ocean, Atmospheric Sciences, University of British Columbia, Vancouver, BC, Canada

**Table of Contents:**

**Page SI-3: Figure SI1**: Chemical structures of **RA-312**, **RA-313**, **RA-314-1/2** and **RA-315**.

**Page SI-4**: **Table SI1.** ^13^C NMR Data for **RA-312, RA-313, RA-314-1, RA-314-2** and **RA-315** recorded at 150 MHz in C_6_D_6_ (δ in ppm).

**Page SI-4**: **Table SI2.** ^1^H NMR Data for **RA-312, RA-313, RA-314-1, RA-314-2** and **RA-315** recorded at 600 MHz in C_6_D_6_ (δ in ppm, *J* in Hz).

**Page SI-5**: ^1^H NMR Spectrum of **RA-313** recorded at 600 MHz in C_6_D_6_.

**Page SI-6**: Expanded ^1^H NMR Spectrum of **RA-313** with peak picking recorded at 600 MHz in C_6_D_6_.

**Page SI-7**: Expanded ^1^H NMR Spectrum of **RA-313** with peak picking recorded at 600 MHz in C_6_D_6_.

**Page SI-8**: Expanded ^1^H NMR Spectrum of **RA-313** with peak picking recorded at 600 MHz in C_6_D_6_.

**Page SI-9**: ^13^C NMR Spectrum of Impure **RA-313** recorded at 150 MHz in C_6_D_6_.

**Page SI-10**: gradCOSY NMR Spectrum of **RA-313** recorded at 600 MHz in C_6_D_6_.

**Page SI-11**: gradHSQC NMR Spectrum of **RA-313** recorded at 600 MHz in C_6_D_6_.

**Page SI-12**: gradHMBC NMR Spectrum of **RA-313** recorded at 600 MHz in C_6_D_6_.

**Page SI-13**: tROESY NMR Spectrum of **RA-313** recorded at 600 MHz in C_6_D_6_.

**Page SI-14**: ^1^H NMR Spectrum of **RA-314-1** & **2** recorded at 600 MHz in C_6_D_6_.

**Page SI-15**: Expanded ^1^H NMR Spectrum of **RA-314-1** & **2** with peak picking recorded at 600 MHz in C_6_D_6_.

**Page SI-16**: Expanded ^1^H NMR Spectrum of a Mixture of **RA-314-1** & **2** with peak picking recorded at 600 MHz in C_6_D_6_.

**Page SI-17**: Expanded ^1^H NMR Spectrum of a Mixture of **RA-314-1** & **2** with peak picking recorded at 600 MHz in C_6_D_6_.

**Page SI-18**: ^13^C NMR Spectrum of a Mixture of **RA-314-1** & **2** recorded at 150 MHz in C_6_D_6_.

**Page SI-19**: gradCOSY NMR Spectrum of a Mixture of **RA-314-1** & **2** recorded at 600 MHz in C_6_D_6_.

**Page SI-20**: gradHSQC NMR Spectrum of a Mixture of **RA-314-1** & **2** recorded at 600 MHz in C_6_D_6_.

**Page SI-21**: gradHMBC NMR Spectrum of a Mixture of **RA-314-1** & **2** recorded at 600 MHz in C_6_D_6_.

**Page SI-22**: tROESY NMR Spectrum of a Mixture of **RA-314-1** & **2** recorded at 600 MHz in C_6_D_6_.

**Page SI-23**: ^1^H NMR Spectrum of **RA-315** recorded at 600 MHz in C_6_D_6_.

**Page SI-24**: Expanded ^1^H NMR Spectrum of **RA-315** with peak picking recorded at 600 MHz in C_6_D_6_.

**Page SI-25**: Expanded ^1^H NMR Spectrum of **RA-315** with peak picking recorded at 600 MHz in C_6_D_6_.

**Page SI-26**: Expanded ^1^H NMR Spectrum of **RA-315** with peak picking recorded at 600 MHz in C_6_D_6_.

**Page SI-27**: ^13^C NMR Spectrum of **RA-315** recorded at 150 MHz in C_6_D_6_.

**Page SI-28**: gradCOSY NMR Spectrum of **RA-315** recorded at 600 MHz in C_6_D_6_.

**Page SI-29**: gradHSQC NMR Spectrum of **RA-315** recorded at 600 MHz in C_6_D_6_.

**Page SI-30**: gradHMBC NMR Spectrum of **RA-315** recorded at 600 MHz in C_6_D_6_.

**Page SI-31**: tROESY NMR Spectrum of **RA-315** recorded at 600 MHz in C_6_D_6_.

**Page SI-32**: **Figure SI2**: Sesquiterpene lactones isolated from *A. cordifolia* are cytotoxic to HT-29 cells. HT-29 cells were treated with varying concentrations of RA-312 (a), RA-313 (b), RA-314 (c), or RA-315 (d) for 72 h, then cell viability was determined by MTT assay. Standard errors of the means are shown.

**
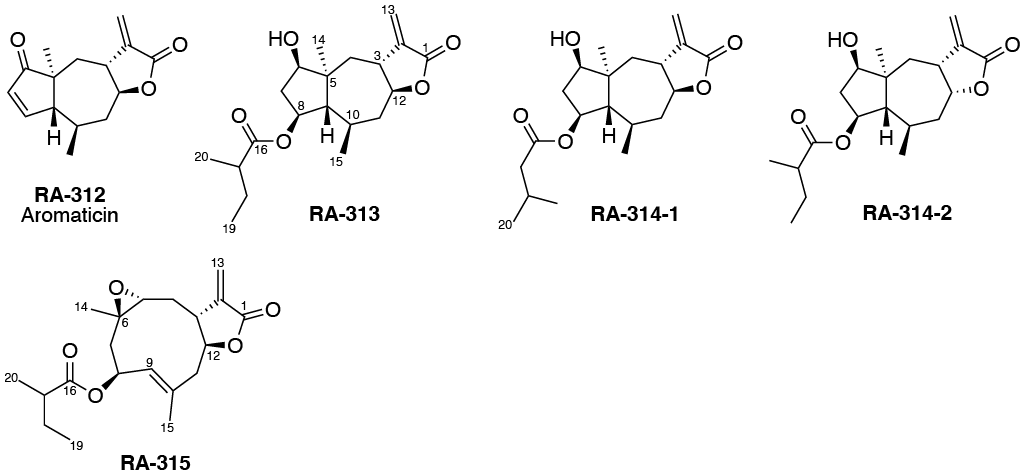
**

**Figure SI1**: Chemical structures of **RA-312**, **RA-313**, **RA-314-1/2** and **RA-315**

**Table SI1.** ^13^C NMR Data for **RA-312, RA-313, RA-314-1,**

**RA-314-2** and **RA-315** recorded at 150 MHz in C_6_D_6_ (δ in ppm)

| C# | **RA-312** | **RA-313** | **RA-314-1** | **RA-314-2** | **RA-315** |
| --- | --- | --- | --- | --- | --- |
| 1 | 168.0, C | 169.2, C | 169.2, C | 169.0, C | 168.1, C |
| 2 | 140.5, C | 141.7, C | 141.6, C | 141.4, C | 140.0, C |
| 3 | 45.2, CH | 45.5, CH | 45.5, CH | 40.8, CH | 45.8, CH |
| 4 | 31.4, CH2 | 33.8, CH_2_ | 33.8, CH_2_ | 39.4, CH_2_ | 30.8, CH_2_ |
| 5 | 49.8, C | 47.2, C | 47.2, C | 46.0, C | 65.7, CH |
| 6 | 211.0, C | 80.3, CH | 80.3, CH | 80.8, CH | 59.3, C |
| 7 | 129.2, CH | 40.7, CH_2_ | 40.7, CH_2_ | 42.4, CH_2_ | 43.7, CH_2_ |
| 8 | 159.4, CH | 77.9, CH | 77.8, CH | 78.8, CH | 68.6, CH |
| 9 | 52.6, CH | 51.0, CH | 51.0, CH | 50.0, CH | 127.9, CH |
| 10 | 26.0, CH | 28.6, CH | 28.6, CH | 29.0, CH | 133.1, C |
| 11 | 43.4, CH_2_ | 44.0, CH_2_ | 44.1, CH_2_ | 35.2, CH_2_ | 46.7, CH_2_ |
| 12 | 77.9, CH | 81.3, CH | 81.4, CH | 76.2, CH | 82.7, CH |
| 13 | 117.8, CH_2_ | 118.1, CH_2_ | 118.2, CH_2_ | 121.0, CH_2_ | 120.4, CH_2_ |
| 14 | 26.7, CH_3_ | 25.3, CH_3_ | 25.3, CH_3_ | 21.7, CH_3_ | 17.1, CH_3_ |
| 15 | 18.6, CH_3_ | 21.1, CH_3_ | 20.9, CH_3_ | 20.6, CH_3_ | 17.9, CH_3_ |
| 16 |  | 175.6, C | 172.2, C | 175.7, C | 175.3, C |
| 17 |  | 41.5, CH | 43.8, CH_2_ | 41.5, CH | 41.2, CH |
| 18 |  | 26.8, CH_2_ | 25.9, CH | 26.8, CH_2_ | 27.1, CH_2_ |
| 19 |  | 11.8, CH_3_ | 22.5, CH_3_^a^ | 11.8, CH_3_ | 11.7, CH_3_ |
| 20 |  | 16.7, CH_3_ | 22.4, CH_3_^a^ | 16.7, CH_3_ | 16.8, CH_3_ |

^a^Assignments within a column are interchangeable.

**Table SI2.** ^1^H NMR Data for **RA-312, RA-313, RA-314-1, RA-314-2** and **RA-315** recorded at 600 MHz in C_6_D_6_ (δ in ppm, *J* in Hz)

| C# | **RA-312** | **RA-313** | **RA-314-1** | **RA-314-2** | **RA-315** |
| --- | --- | --- | --- | --- | --- |
| 3 | 1.92, m | 2.24^a^ | 2.29^a^ | 3.02^a^ | 1.91, m |
| 4 | 1.16, dd (14.2, 11.0)  2.00, dd (14,2, 7.1) | 0.56, dd (13.8,11.9)  2.45, dd (13.9, 5.3) | 0.56, dd (13.7,11.8)  2.44, dd (13.7, 5.2) | 0.99, t (13.8)  1.61, dd (14.7, 4.0) | 0.73^a^  1.80^a^ |
| 5 |  |  |  |  | 2.10, bd (10.1) |
| 6 |  | 3.09, bt (4.4) | 3.10, bt (3.4) | 3.02^a^ |  |
| 7 | 5.75, dd (6,0, 2.9) | 1.25, dd (15.7, 2.3)  2.29, ddd (15.6, 9.4, 4.8) | 1.29, dd (15.7, 2.4)  2.29^a^ | 1.19, dd (15.8, 3.2)  2.42, ddd (15.9, 9.2, 5.2) | 1.28, t (11.5)  2.43, dd (12.0, 5.8) |
| 8 | 6.72, dd (6.0, 1.8) | 4.84, ddd (9.6, 7.8, 2.3) | 4.86, ddd (9.2, 7.8, 2.2) | 4.67, td (8.8, 3.1) | 5.54, ddd (10.8, 10.8, 5.9) |
| 9 | 1.74, ddd (10.4, 2.2, 2.2) | 2.04, dd (10.7, 7.6) | 2.06, dd (10.7, 7.6) | 1.83, dd (11.8, 8.2) | 4.82, bd (10.5) |
| 10 | 1.22, m | 1.25^a^ | 1.26^a^ | 1.35^a^ |  |
| 11 | 0.70, ddd (12.4, 12.4, 12.4)  1.88, ddd (12.8, 4.3, 3.2) | 0.91, ddd (12.3, 12.3, 12.3)  1.90, dt (12.8, 3.5) | 0.94, ddd (12.4, 12.4, 12.4)  1.91, dt (12.8, 3.6) | 1.48, ddd (13.7, 3.4, 1.4)  1.59^a^ | 1.80^a^  2.35, bd (12.4) |
| 12 | 3.68, ddd (11.6, 9.5, 3.2) | 3.50, ddd (11.8, 9.3, 3.2) | 3.51, ddd (11.7, 9.2, 3.2) | 4.12, ddd (12.4, 7.9, 3.4) | 3.19, ddd (10.8, 7.5, 1.5) |
| 13 | 4.85, d (3.2)  6.04 d (3.5) | 4.94, d (3.0)  6.10, d (3.5) | 4.95, d (3.2)  6.10, d (3.5) | 5.14, bd (2.2)  6.22, bd (2.4) | 4.86, d (3.1)  6.16, d (3.6) |
| 14 | 0.64, s | 0.25, s | 0.26, s | 0.27, s | 0.73, s |
| 15 | 0.55, d (6.5) | 0.80, d (6.7) | 0.81, d (6.8) | 0.85, d (6.4) | 1.48, d (1.1) |
| 17 |  | 2.24^a^ | 2.03, m | 2.24, qt (6.8, 6.8) | 2.26, qdd (6.7, 6.7, 6.7) |
| 18 |  | 1.34, m  1.70, m | 2.12, qqt (6.9, 6.9, 6.9) | 1.35^a^  1.70, dqd (13.8, 7.4, 7.4) | 1.35, m  1.65, dqd (13.6, 7.4, 7.4) |
| 19 |  | 0.84, t (7.5) | 0.87, d (6.7)^b^ | 0.84, t (7.4) | 0.82, t (7.4) |
| 20 |  | 1.07, d (7.0) | 0.86, d (6.6)^b^ | 1.07, d (7.0) | 1.07, d (7.0) |
| 6-OH |  | 1.11, bd (4.5) | 1.12, bd (3.3) | 1.04, bs |  |

^a^Multiplicity not determined due to overlapping signals - chemical shifts determined from 2D data.

^b^Assignments within a column are interchangeable.

^1^H NMR Spectrum of **RA-313** recorded at 600 MHz in C_6_D_6_

_
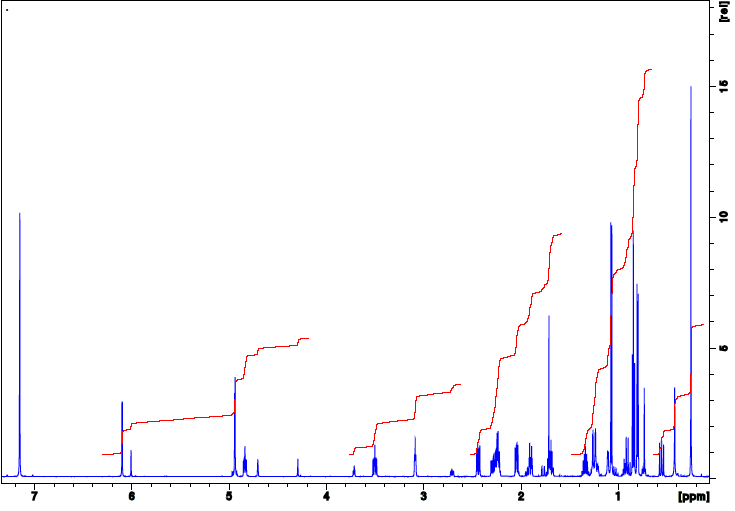
_

__

Expanded ^1^H NMR Spectrum of **RA-313** with peak picking recorded at 600 MHz in C_6_D_6_

_
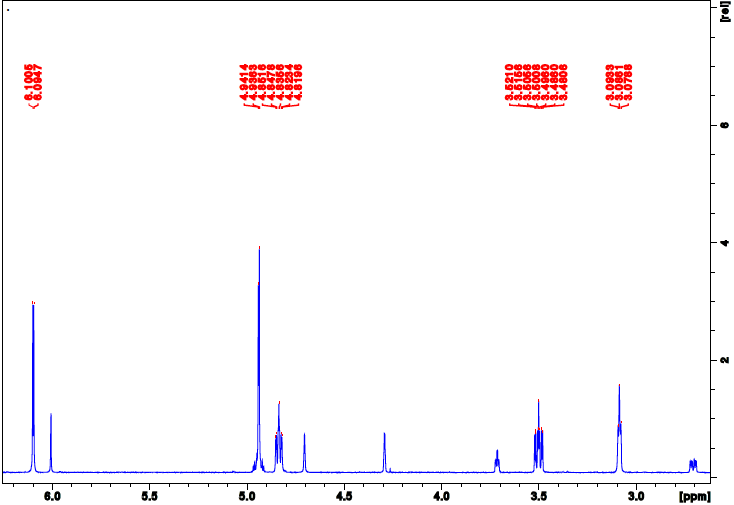
_

__

Expanded ^1^H NMR Spectrum of **RA-313** with peak picking recorded at 600 MHz in C_6_D_6_

_
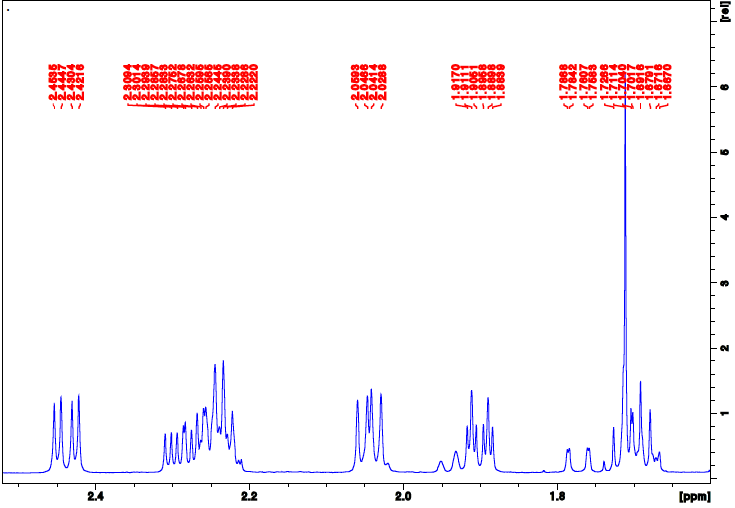
_

__

Expanded ^1^H NMR Spectrum of **RA-313** with peak picking recorded at 600 MHz in C_6_D_6_

_
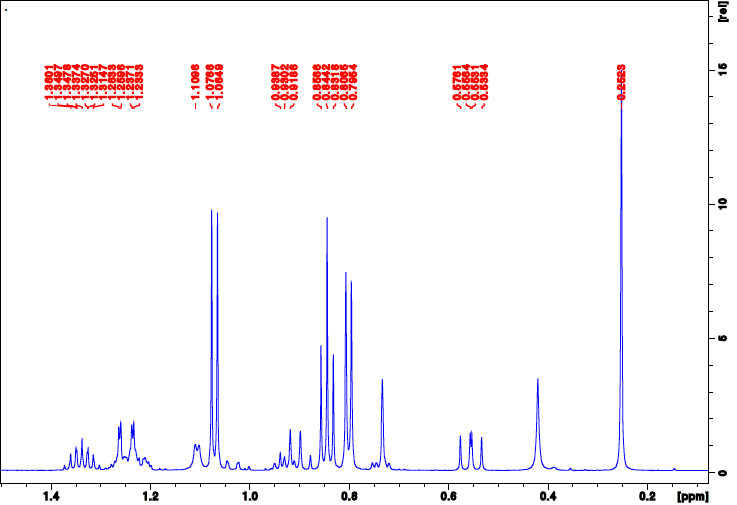
_

__

^13^C NMR Spectrum of **RA-313** recorded at 150 MHz in C_6_D_6_

_
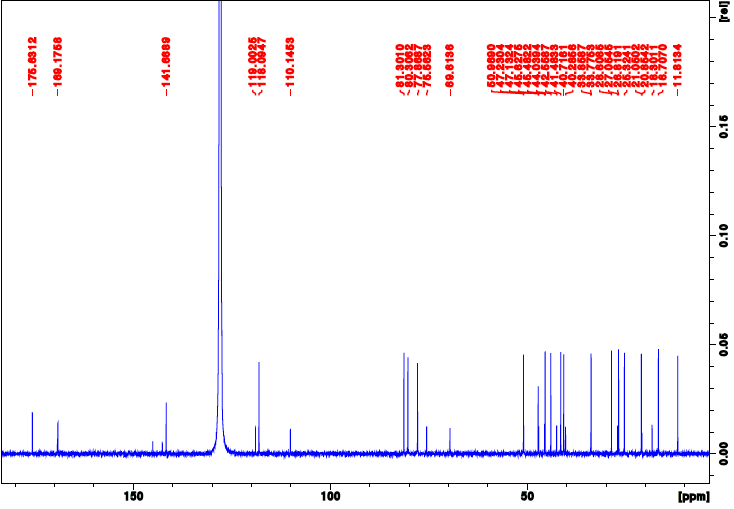
_

__

gradCOSY NMR Spectrum of **RA-313** recorded at 600 MHz in C_6_D_6_

_
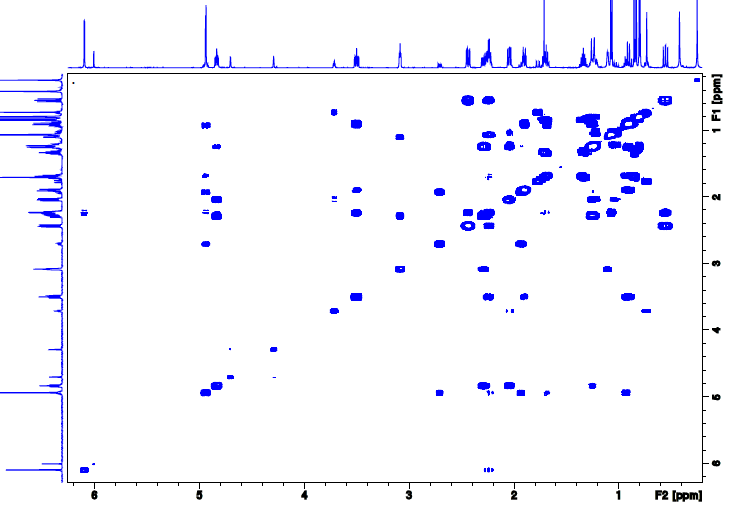
_

__

gradHSQC NMR Spectrum of **RA-313** recorded at 600 MHz in C_6_D_6_

_
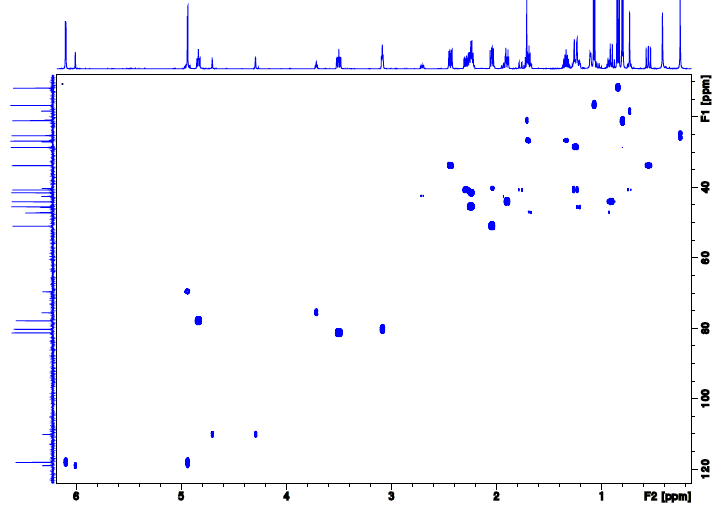
_

__

gradHMBC NMR Spectrum of **RA-313** recorded at 600 MHz in C_6_D_6_

_
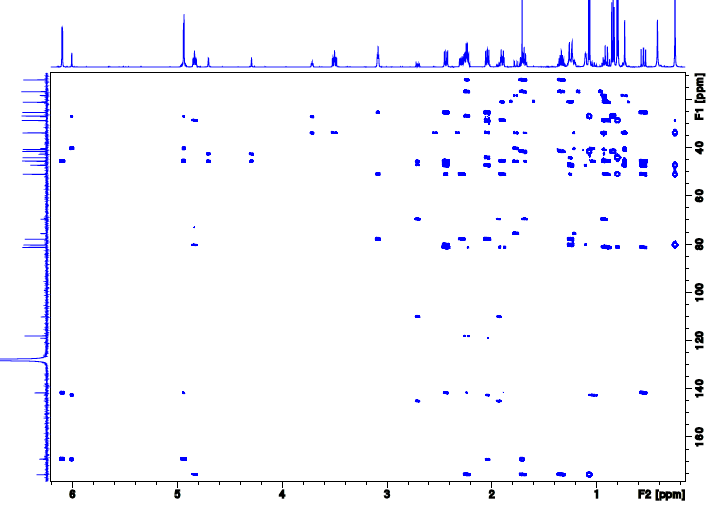
_

__

tROESY NMR Spectrum of **RA-313** recorded at 600 MHz in C_6_D_6_

_
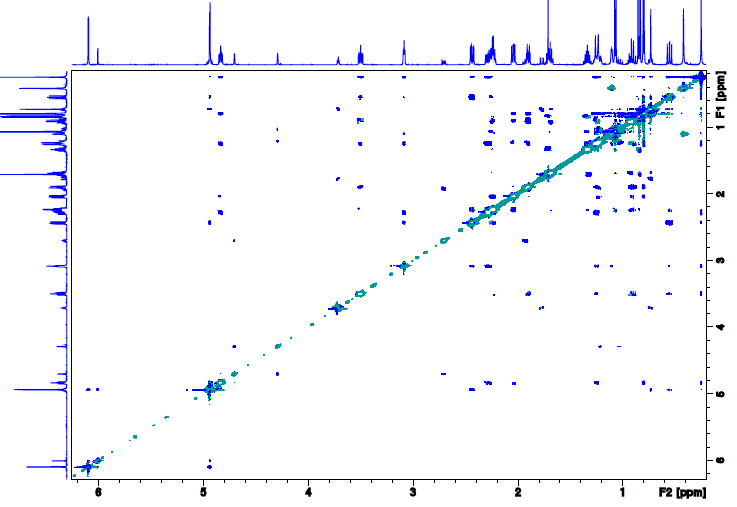
_

__

^1^H NMR Spectrum of a Mixture of **RA-314-1** & **2** recorded at 600 MHz in C_6_D_6_


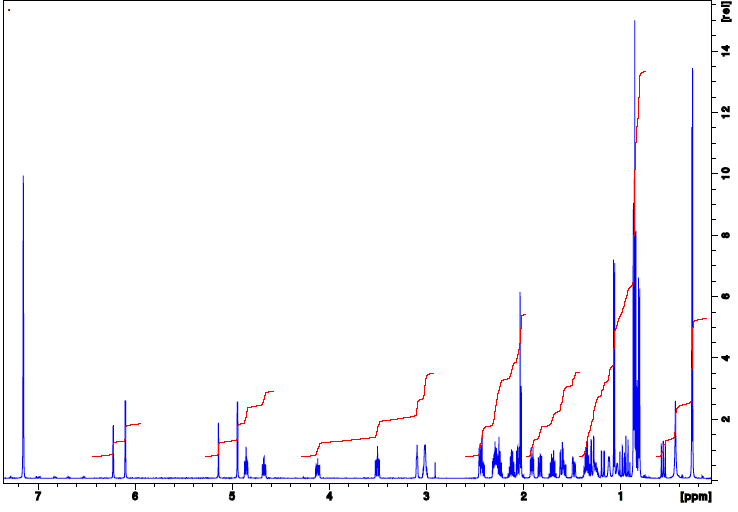

Expanded ^1^H NMR Spectrum of a Mixture of **RA-314-1** & **2** with peak picking recorded at 600 MHz in C_6_D_6_


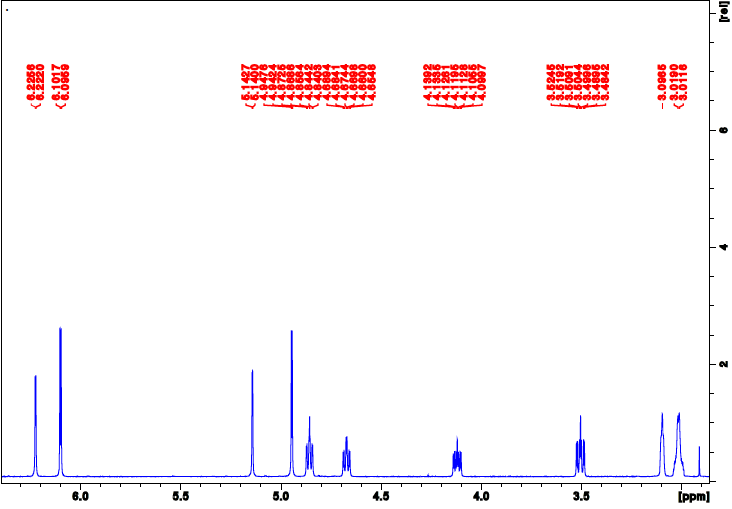

Expanded ^1^H NMR Spectrum of a Mixture of **RA-314-1** & **2** with peak picking recorded at 600 MHz in C_6_D_6_


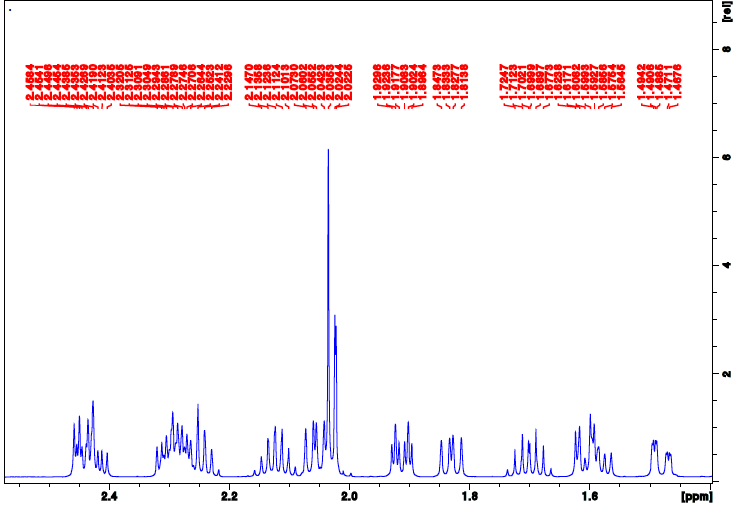

Expanded ^1^H NMR Spectrum of a Mixture of **RA-314-1** & **2** with peak picking recorded at 600 MHz in C_6_D_6_

_
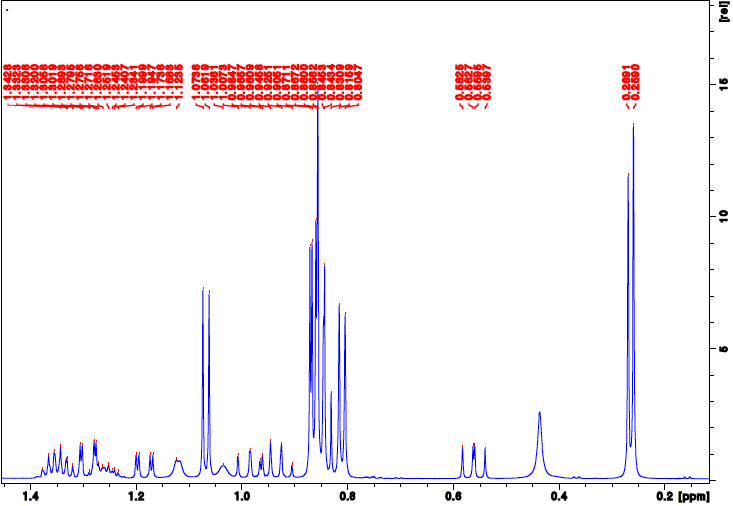
_

^13^C NMR Spectrum of a Mixture of **RA-314-1** & **2** recorded at 150 MHz in C_6_D_6_


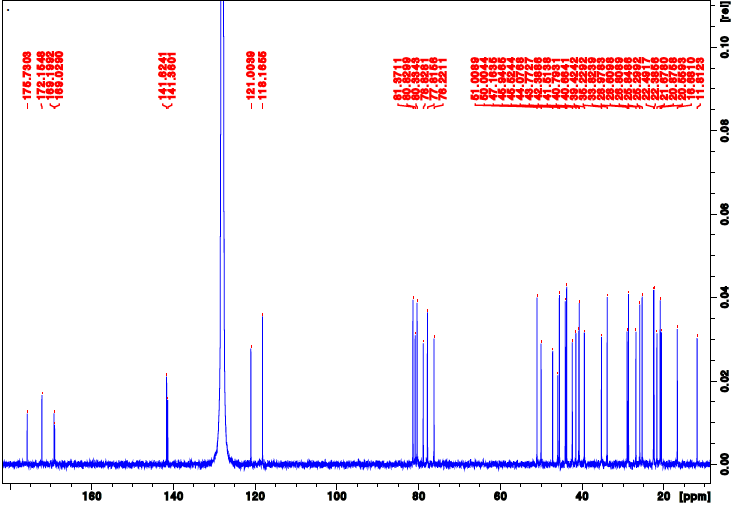

gradCOSY NMR Spectrum of a Mixture of **RA-314-1** & **2** recorded at 600 MHz in C_6_D_6_


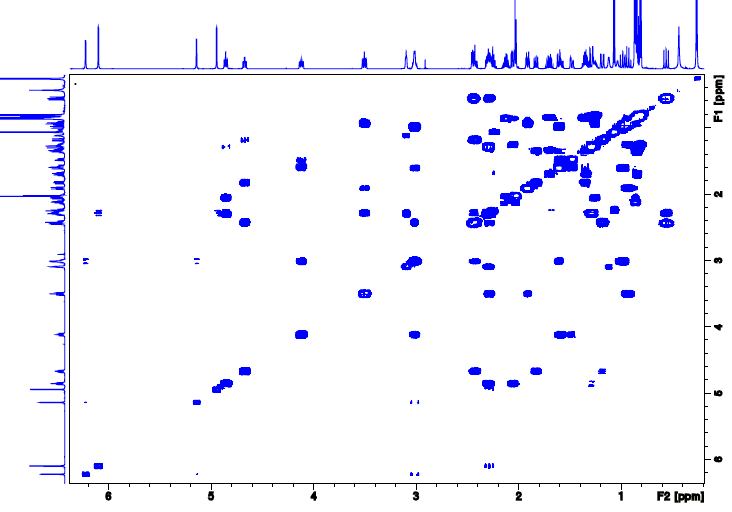

gradHSQC NMR Spectrum of a Mixture of **RA-314-1** & **2** recorded at 600 MHz in C_6_D_6_


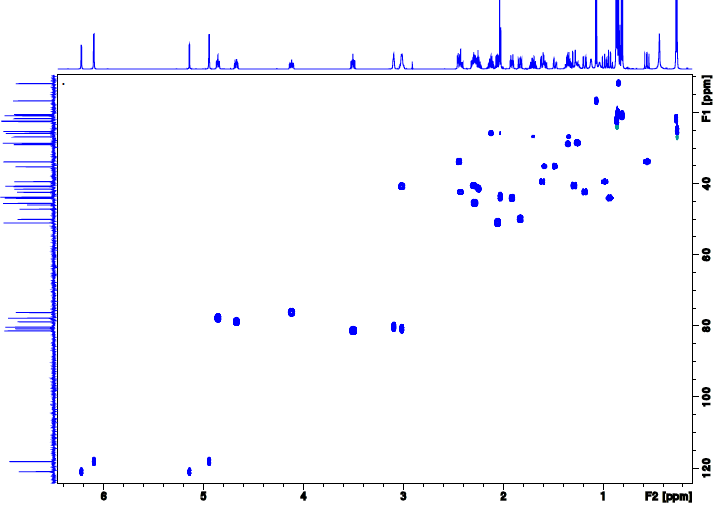

gradHMBC NMR Spectrum of a Mixture of **RA-314-1** & **2** recorded at 600 MHz in C_6_D_6_

_
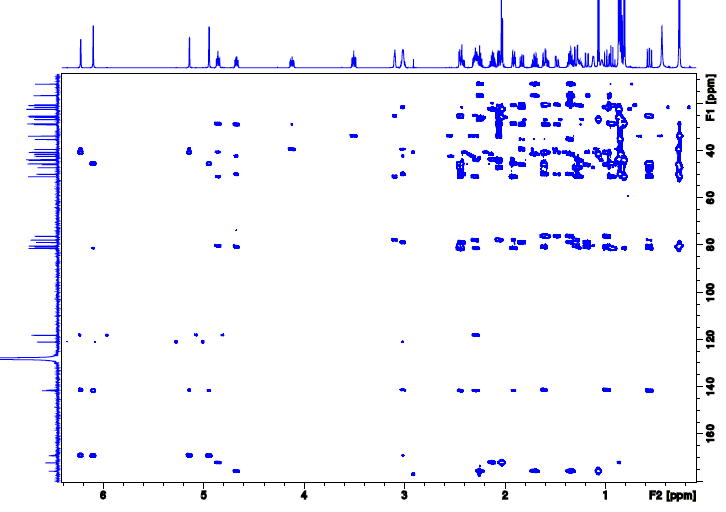
_

tROESY NMR Spectrum of a Mixture of **RA-314-1** & **2** recorded at 600 MHz in C_6_D_6_


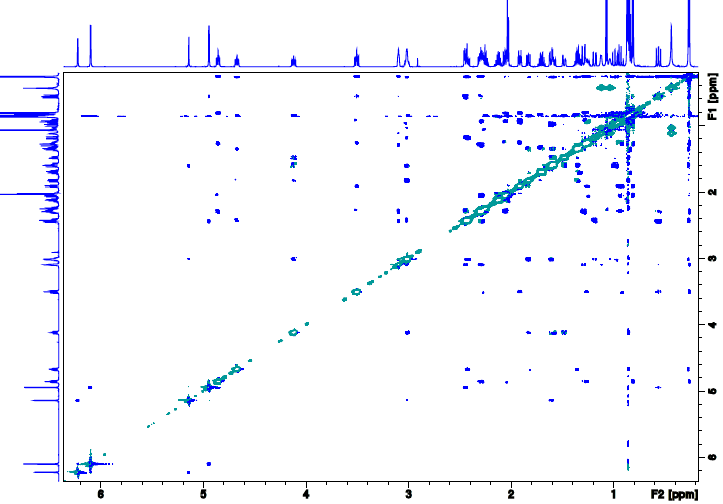

^1^H NMR Spectrum of **RA-315** at 600 MHz in C_6_D_6_

_
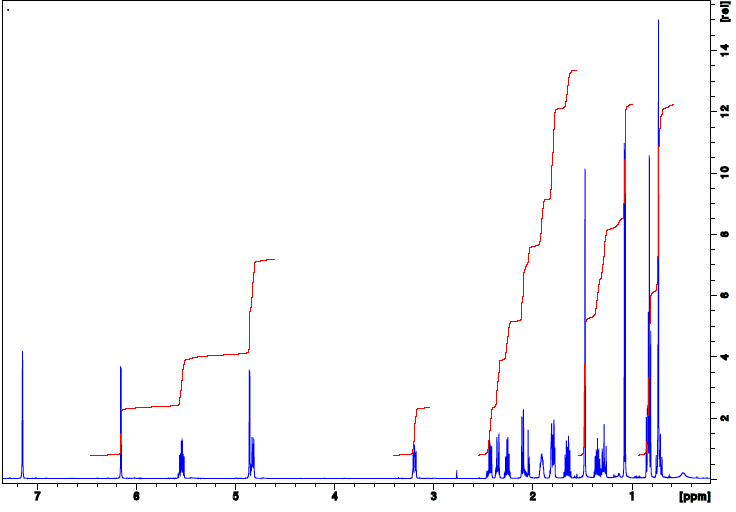
_

Expanded ^1^H NMR Spectrum of **RA-315** with peak picking recorded at 600 MHz in C_6_D_6_


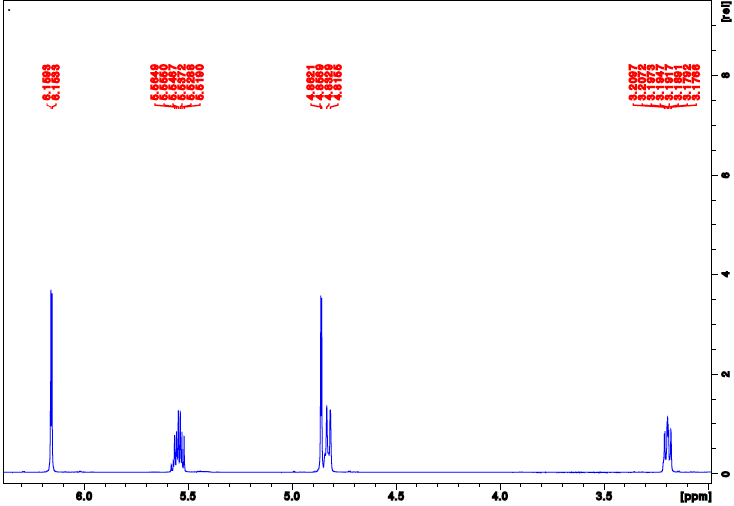

Expanded ^1^H NMR Spectrum of **RA-315** with peak picking recorded at 600 MHz in C_6_D_6_


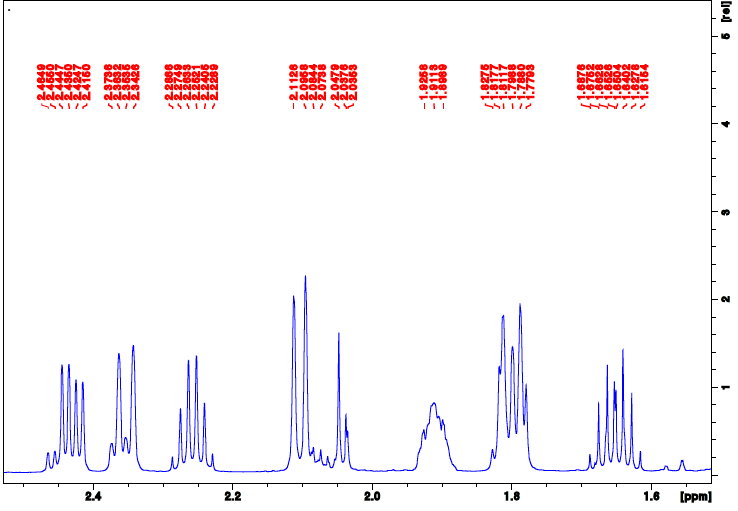

Expanded ^1^H NMR Spectrum of **RA-315** with peak picking recorded at 600 MHz in C_6_D_6_

_
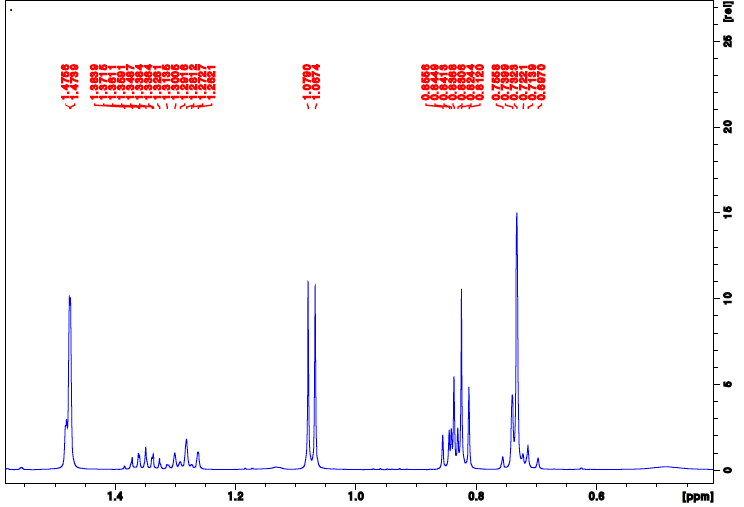
_

^13^C NMR Spectrum of **RA-315** recorded at 150 MHz in C_6_D_6_


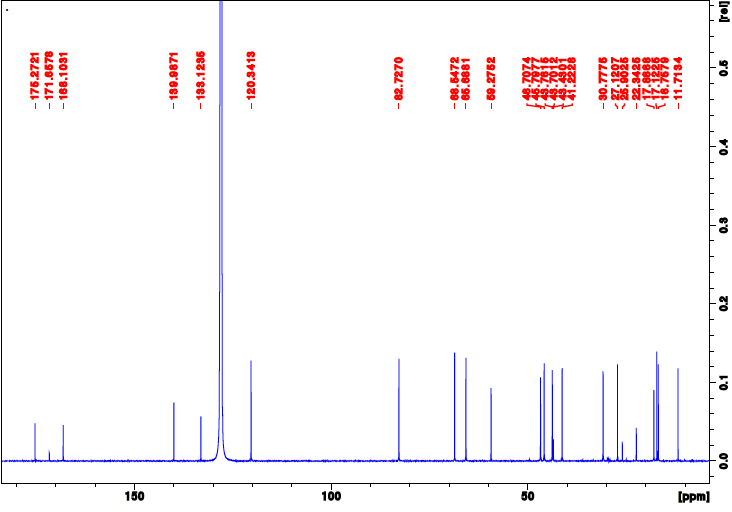

gradCOSY NMR Spectrum of **RA-315** recorded at 600 MHz in C_6_D_6_


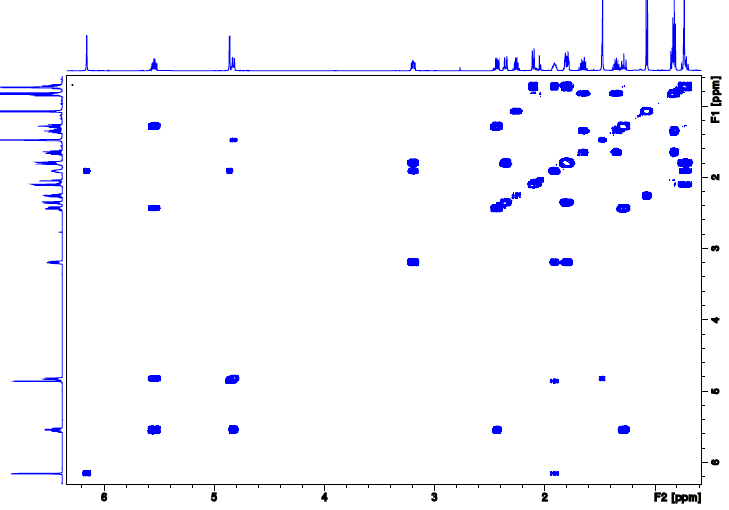

gradHSQC NMR Spectrum of **RA-315** recorded at 600 MHz in C_6_D_6_


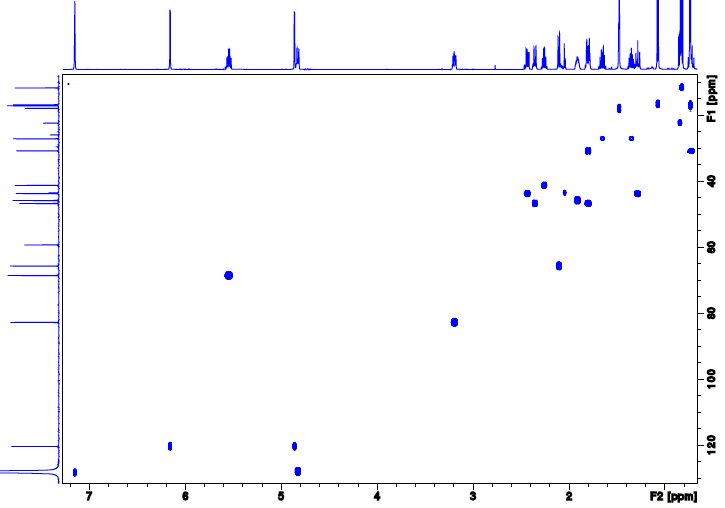

gradHMBC NMR Spectrum of **RA-315** recorded at 600 MHz in C_6_D_6_


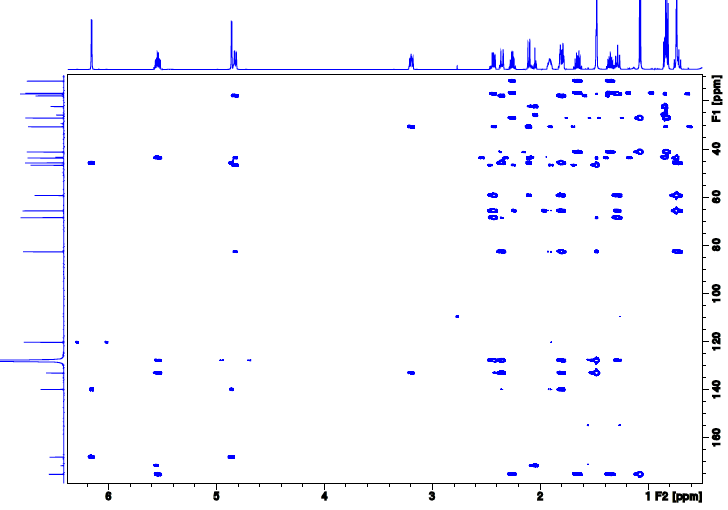

tROESY NMR Spectrum of **RA-315** recorded at 600 MHz in C_6_D_6_


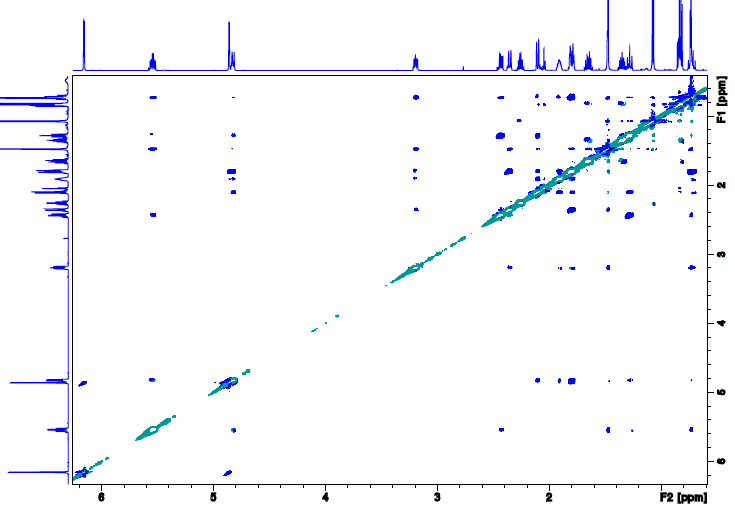

**
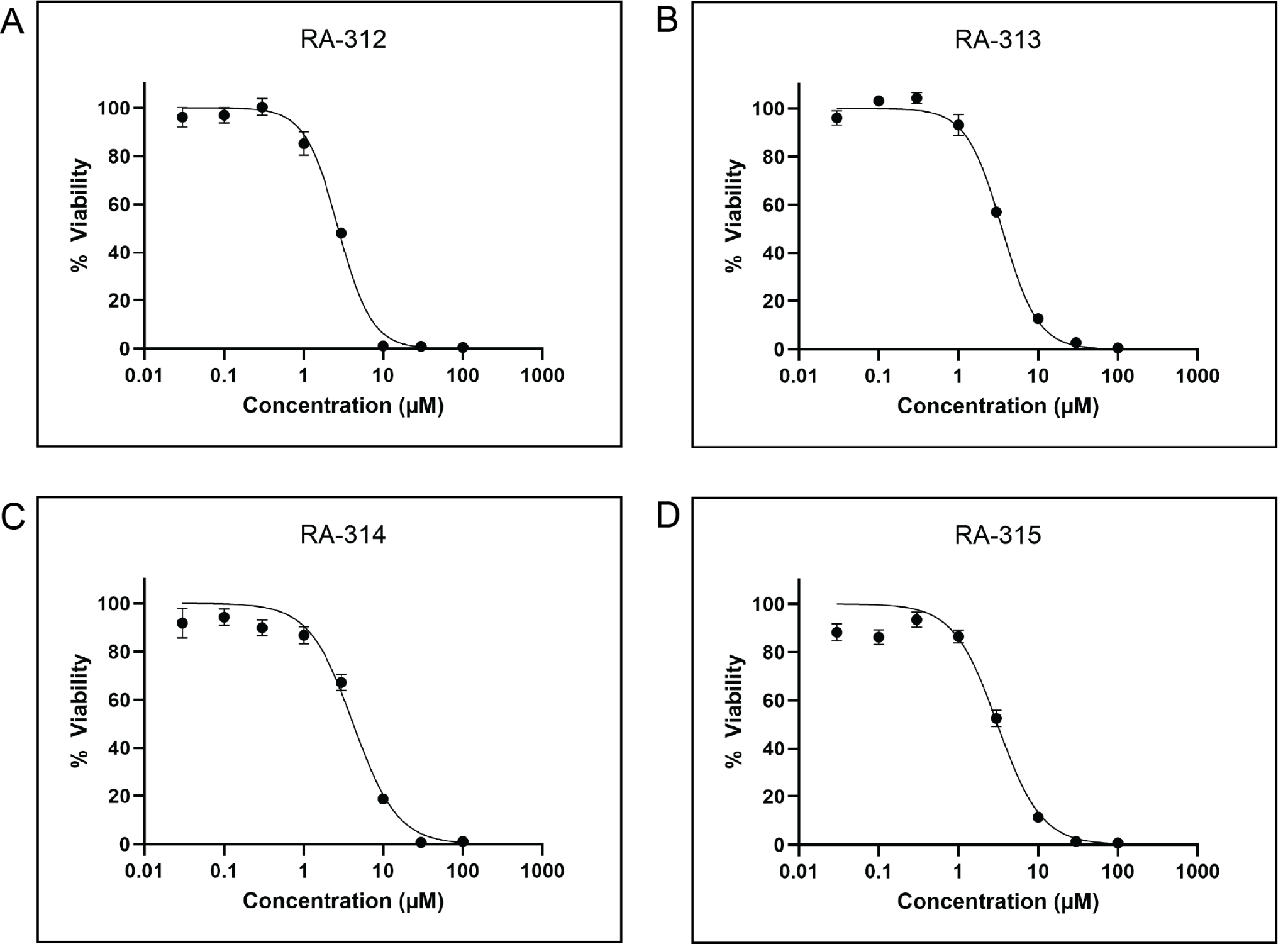
**

**Figure SI2.** Sesquiterpene lactones isolated from *A. cordifolia* are cytotoxic to HT-29 cells. HT-29 cells were treated with varying concentrations of RA-312 (a), RA-313 (b), RA-314 (c), or RA-315 (d) for 72 h, then cell viability was determined by MTT assay. Standard errors of the means are shown.
